# Genome-wide identification of WRKY members in elephant grass (*Cenchrus purpureus*) and its expression profiling under aluminum stress

**DOI:** 10.7717/peerj.21299

**Published:** 2026-06-04

**Authors:** Mengying Ruan, Zhe Ma, Fuqiang Li, Wenyi Zhao, Nini Hu, Han Zhou, Chengxi Zhang, Qiang Zhou, Yuguo Wu

**Affiliations:** 1 College of Animal Science and Technology, Guangxi Key Laboratory of Animal Breeding, Disease Control and Prevention, Guangxi Grass Station, Guangxi University, Nanning, Guangxi, China; 2 State Key Laboratory of Grassland Agro-ecosystems, Key Laboratory of Grassland Livestock Industry Innovation, Ministry of Agriculture and Rural Affairs, Engineering Research Center of Grassland Industry, Ministry of Education, College of Pastoral Agriculture Science and Technology, Lanzhou University, Lanzhou, Gansu, China

**Keywords:** Elephant grass, WRKY transcription factor, Genome-wide analysis, Aluminum stress, Expression profiles

## Abstract

Elephant grass (*Cenchrus purpureus*) shows strong aluminum (Al) tolerance, and studying its mechanisms could provide gene resources for the improvement of Al-sensitive species. WRKY transcription factors (TFs) have been extensively studied for their roles in Al stress response, yet their specific functions in elephant grass under Al stress remain uncharacterized. In this study, we identified 174 CpWRKY TFs in elephant grass, among which 170 were mapped to 14 chromosomes, and four to unanchored contigs. Phylogenetic analysis revealed that they were classified into seven subgroups. Gene structures, conserved motifs, and domain distributions showed high similarity within each subgroup, indicating potential functional conservation. Collinearity analysis indicated that segmental duplication was the primary driver of the expansion of the CpWRKY family relative to tandem duplication. During the 24-h Al treatment time-course, transcriptome analysis identified 48 * CpWRKY* genes that were differentially expressed at least at one time point. Promoter analysis revealed that stress-responsive elements were the most abundant compared with other categories. Protein-DNA and protein-protein interaction network analyses indicated that WRKY TFs in elephant grass may mediate responses to Al stress through antioxidant defense system, organic acid secretion, and hormone signaling pathways. Overall, this work characterizes the CpWRKY TFs in elephant grass and reveals their potential roles in Al response.

## Introduction

Acidic soils (pH < 5.5) account for approximately 30% of the Earth’s ice-free land surface and are most commonly distributed in humid tropical and subtropical regions (Shen & Zhao, 2019). Acidic soils are often associated with the presence of soluble aluminum (Al), and its toxicity is a key factor limiting crop production by inhibiting root elongation and cell division in the root apical meristem (Kochian et al., 2015). In China, acidic Al soils are mainly distributed across the red soil regions of 14 provinces (municipalities and autonomous regions), covering an area of 2.18 million km^2^, which accounts for 22.7% of the country’s total land area (Shen & Zhao, 2019). Studying plant Al tolerance mechanisms can not only provide new insights into theoretical understanding but also offer genetic resources for molecular breeding aimed at improving Al tolerance.

Over the course of evolution, plants have evolved diverse strategies to withstand Al toxicity, with external exclusion and internal tolerance identified as the two primary adaptive mechanisms (Kochian et al., 2015). The mechanisms of Al exclusion involve the release of organic acids; the secretion of benzoxazinoids and phenolic compounds; enhanced release of mucilage from the root cap and border cells; regulation of rhizosphere pH; and modification of the cell wall, all contributing to external exclusion of Al^3+^ (Ofoe et al., 2023). In wheat (*Triticum aestivum*), the Al-responsive malate transporter (ALMT) gene *TaALMT1* encodes a channel in the plasma membrane that facilitates malate release under Al^3^^+^ stress (Sasaki et al., 2004). Likewise, in barley (*Hordeum vulgare*), the Al-induced citrate transporter HvAACT1 is also targeted to the plasma membrane of root tip cells, where it promotes citrate extrusion and contributes to organic acid-mediated Al detoxification (Furukawa et al., 2007). In contrast, the internal detoxification mechanisms of Al in plants can be summarized as follows: vacuolar sequestration, antioxidant defense, and hormone signal regulation, collectively contributing to the safe compartmentalization of Al^3+^ (Ofoe et al., 2023). The ABC transporter Al Sensitive 1 (ALS1), localized to the vacuolar membrane in rice (*Oryza sativa*), facilitates vacuolar sequestration of Al^3+^ and promotes intracellular detoxification (Ofoe et al., 2023). In buckwheat, the iron-regulated/iron transporter (IREG) protein FeIREG1 is localized to the vacuole, where it contributes to internal Al tolerance by transporting Al^3^^+^ into root vacuoles (Yokosho et al., 2016). Similarly, the soybean homolog (GmIREG3) exhibits a similar function, and overexpression of these *IREG* genes in Arabidopsis (*Arabidopsis thaliana*) has been shown to improve Al tolerance (Cai et al., 2020). These genes are typically targeted and bound by transcription factors (TFs), which regulate their expression.

TFs play crucial roles in Al response mechanisms, including MYBs, C2H2s, bHLHs, and WRKYs (Liu et al., 2022; Huang et al., 2018; Shu et al., 2022). The earliest discovery of WRKY TFs dates back to 1994 in sweet potato (*Ipomoea batatas*), and they are named after the highly conserved “WRKYGQK” amino acid sequence present in the protein structure of members of this family (Ishiguro & Nakamura, 1994). WRKY proteins bind to the W-box promoter elements *via* the conserved WRKYGQK motif, thereby activating or repressing downstream genes (Rushton et al., 1996). WRKY TFs alleviate the phytotoxic effects of Al stress through external exclusion, internal detoxification, or an integrated combination of these mechanisms. OsWRKY22 can promote citrate secretion by activating Ferric Reductase Defective-like 4 (*OsFRDL4*), thereby participating in the exudation mechanism by which plants respond to Al stress (Li et al., 2018). GmWRKY81 can be induced by Al stress; its overexpression enhances Al tolerance in soybean, possibly by influencing the expression of Al transporter and antioxidant-related genes (Shu et al., 2022). In soybean, GmWRKY21 enhances Al tolerance by upregulating organic acid exudation and antioxidant enzyme activity, thereby contributing to both external exclusion and internal detoxification mechanisms (Han et al., 2022).

As a perennial C_4_ forage, elephant grass (*Cenchrus purpureus*) combines fast growth, strong adaptability, and high biomass production (Yan et al., 2021; Hu et al., 2025). It has diverse applications, including the production of bio-based compounds, soil conservation, and bioenergy (Ishii et al., 2015). Elephant grass is widely distributed in subtropical-tropical regions that overlap with acidic Al soils. Some genotypes of elephant grass have also shown a strong capacity to rehabilitate soils affected by heavy metals, including those containing Al (Yan et al., 2021; Deng et al., 2023). The WRKY gene family and its regulatory responses to cold stress have been well characterized in elephant grass (Mao et al., 2024), yet the molecular responses of its WRKY genes to Al stress remain largely unclear. Therefore, elucidating the Al tolerance mechanisms in elephant grass offers a unique opportunity to uncover novel regulatory networks in a highly adaptable, heavy-metal-tolerant species. With the recent release of the elephant grass whole-genome sequence, important resources are now available for identifying Al-responsive TFs. In this study, we conducted a comprehensive analysis of the CpWRKY TFs, including member identification, chromosomal localization, phylogenetic classification, gene structure, and conserved motifs analyses. By integrating protein structure modeling and synteny analysis to explore evolutionary relationships, together with analyses of expression patterns across tissues and developmental stages, *cis*-regulatory element prediction, RT-qPCR assays, and gene expression correlation analyses, the functional mechanisms were elucidated. Finally, protein-DNA interaction (PDI) and protein-protein interaction (PPI) networks, along with Gene Ontology/Kyoto Encyclopedia of Genes and Genomes (GO/KEGG) enrichment analyses of the predicted downstream target genes, were constructed, revealing the putative regulatory pathways of CpWRKY TFs. This study enhances knowledge of Al-responsive transcriptional regulation in Al-tolerant forage species and provides gene resources for improving Al-sensitive species.

## Materials & Methods

### Identification WRKY members of elephant grass

Elephant grass genomic data were retrieved *via* the China National Center for Bioinformation (https://www.cncb.ac.cn/), assigned identifier GWHAORA00000000 (Yan et al., 2021). The protein sequences of WRKY TFs from three reference plants—Arabidopsis, rice (*Oryza sativa*), and maize (*Zea mays*)—were obtained from the Plant Transcription Factor Database (https://planttfdb.gao-lab.org/) (Jin et al., 2017), with detailed locus information provided in Table S1. Furthermore, the hidden Markov model (HMM) profile representing the WRKY DNA-binding domain (PF03106) was acquired from the Pfam database (http://pfam-legacy.xfam.org/) (Mistry et al., 2021). First, BLASTp was used to align the elephant grass genome with the WRKY members of three reference species, resulting in the identification of candidate *WRKY* genes in elephant grass. Then, the simple HMM search function in TBtools-II (v2.147) software was used to filter and validate the conserved domains of the candidate genes, restricting E-values to less than 10^−^^5^ to maintain prediction accuracy (Chen et al., 2023). Conserved domains were further verified using the NCBI Conserved Domain search (CD-Search) tool (https://www.ncbi.nlm.nih.gov/Structure/cdd/) (Lu et al., 2020) and the InterPro database (https://www.ebi.ac.uk/interpro/) (Paysan-Lafosse et al., 2023). Following the removal of redundant or incomplete sequences, the final set of CpWRKY TFs was established.

Moreover, the “Protein parameter Calc” in the TBtools-II software (v2.147) was utilized to conduct detailed predictions on the physical and chemical properties of the relevant proteins in elephant grass, covering important indicators such as the number of amino acids, molecular weight, and isoelectric point. Finally, the WoLF PSORT (https://wolfpsort.hgc.jp/) website was adopted to predict the subcellular localization of the CpWRKY TFs in elephant grass (Horton et al., 2007).

### Chromosomal distribution, phylogenetic analysis, and classification of CpWRKYs

The chromosome location and length data of *CpWRKY* genes were extracted from the GFF file using TBtools, and finally, their position information was visualized with MapGene2Chrom (http://mg2c.iask.in/mg2c_v2.0/) (Chao et al., 2021). A phylogenetic tree was generated using MEGA 7.0 (v7.0.26) to explore the evolutionary relationships between WRKY proteins from Arabidopsis and elephant grass (Kumar, Stecher & Tamura, 2016). CpWRKY and AtWRKY protein sequences were aligned with ClustalW in MEGA 7.0 (v7.0.26), and a neighbor-joining (NJ) tree was constructed using the Poisson model with pairwise gap deletion and 1,000 bootstrap replicates. Visualization and modification of the phylogenetic tree were performed with iTOL (https://itol.embl.de) (Letunic & Bork, 2024).

### Motif analysis, domain mapping, and gene structure of CpWRKYs

The structural visualization of *CpWRKY* genes was conducted using TBtools-II (v2.147). The MEME suite (http://meme-suite.org/tools/meme) was used to examine the motif organization of CpWRKY proteins, setting the number of motifs to 10 while retaining default parameters for other options (Bailey et al., 2015). Conserved domains were characterized using the CDD website. The visual integration of conserved motifs, domain locations, and exon–intron structures was achieved through the Gene Structure Viewer module in TBtools-II (v2.147).

### Secondary structure and 3D model analysis of CpWRKY proteins

Three-dimensional (3D) structures of CpWRKY proteins were modeled using SWISS-MODEL (https://swissmodel.expasy.org/), and model reliability was evaluated by the Global Model Quality Estimation (GMQE) index (Waterhouse et al., 2018). The secondary structures of CpWRKYs were predicted and analysed using SOPMA (https://npsa.lyon.inserm.fr/cgi-bin/npsa_automat.pl?page=/NPSA/npsa_sopma.html) (Geourjon & Deléage, 1995).

### Collinearity and evolutionary selection of *CpWRKY* gene family

Tandem duplication and collinearity relationships among *CpWRKY* gene pairs were examined using the MCScanX module embedded in TBtools-II (v2.147). The same approach was also applied to assess the syntenic relationships between elephant grass and four representative plant species—Arabidopsis, rice, maize, and sorghum (*Sorghum bicolor*). Genome sequences and GFF files for Arabidopsis were retrieved from the TAIR database, while those for rice, maize, and sorghum were downloaded from NCBI. In addition, the “Simple Ka/Ks Calculator (NG)” function in TBtools was applied to evaluate the synonymous substitution rate (Ks), nonsynonymous substitution rate (Ka), and evolutionary selection pressure (Ka/Ks ratio) for the CpWRKY TF family of elephant grass, with the results presented using Origin 2021.

### Gene expression profiles across different tissues and under Al stress

Expression data for CpWRKY TFs across five tissues (root, stem, stem tip, leaf, and flower) were retrieved from the Milletdb database (http://milletdb.novogene.com/tools/geneexpression/) (Sun et al., 2023). In the data analysis stage, we applied a dual selection criterion, the transcripts per million (TPM) value of the target gene in a given tissue must meet two conditions: “greater than twice the average TPM of the other four tissues” and “TPM value >10”. After selecting the differentially highly expressed genes across the five tissues based on this criterion, we further chose the top two genes with the highest fold change in each tissue for visualization of their expression characteristics. For the analysis of expression levels under Al treatment at different time points, root and shoot tissues were first sampled separately at four time points (0, 4, 8, and 24 h) following treatment with one mmol/L AlCl_3_⋅ 6H_2_O, and RNA sequencing (RNA-seq) was then performed. Upregulated genes refer to those with higher expression levels after 24 h of Al treatment compared to the CK group, while downregulated genes are those with lower expression levels after 24 h of Al treatment compared to the CK group. Expression data were log_2_-transformed and then standardized by Z-score transformation to compare gene expression across tissues and Al treatment points. The results were visualized as a heatmap using TBtools-II (v2.147).

### Promoter *cis*-regulatory elements analysis of the CpWRKY family

The 2,000 bp upstream promoter sequences of 48 differentially expressed genes (DEGs) were obtained using the GFF3 Sequence Extraction function in TBtools-II (v2.147). These promoter regions were subsequently analyzed for *cis*-regulatory elements through the PlantCARE database (https://bioinformatics.psb.ugent.be/webtools/plantcare/html/) (Lescot et al., 2002). Subsequently, *cis*-regulatory elements with corresponding functional characteristics were screened and quantified. Heatmap representation of the results was generated using TBtools-II (v2.147) and GraphPad Prism 8 (v8.0.1).

### Protein-DNA and protein-protein interaction network analysis

The AGRIS database (https://agris-knowledgebase.org/regcollection) was used to retrieve potential target genes (Palaniswamy et al., 2006), and a PDI network was generated and visualized in Cytoscape (v3.10.2). Due to the lack of elephant grass-specific data, interactions were predicted based on homologous AtWRKY proteins from Arabidopsis. The GO/KEGG analyses of downstream genes were also performed and presented on the Novogene Magic Platform (https://magic-plus.novogene.com/#). For GO and KEGG enrichment analyses, adjusted *P*-values (padj) < 0.05 were used as the threshold for significant enrichment, and multiple testing correction was performed using the Benjamini–Hochberg (BH) method. The number of W-box elements present in the downstream genes was predicted using the website (https://plantpan.itps.ncku.edu.tw/plantpan4/gene_group_setting.php), with a threshold of support ≥ 100% set as the filtering criterion (Wen et al., 2008). Additionally, PPI analysis among the 48 CpWRKY TFs were inferred through the STRING database (http://string-db.org) (Szklarczyk et al., 2025).

### Plant material

Plant materials of elephant grass (‘Purple’) were obtained from the Guangxi Institute of Animal Husbandry, and subsequent seed germination and cultivation were carried out at Guangxi University. The seeds were alternately washed with 75% ethanol and distilled water three times, and then germinated in Petri dishes. Three days after germination, elephant grass seedlings were hydroponically grown in 1/4 Hoagland nutrient solution (pH 5.8). The nutrient solution was replaced every three days. The 30-day-old elephant grass was subjected to Al treatment. To mimic the bioavailable Al levels (0.05–1 mmol/L) typically found in the acidic soils of southern China (Zhong et al., 2008), we adopted a moderate concentration gradient (Yan et al., 2024) and set one mmol/L AlCl_3_⋅ 6H_2_O as the Al treatment concentration in this research. One day before treatment, elephant grass seedlings were pretreated with 0.5 mmol/L CaCl_2_ (pH 4.5), and then exposed to 0.5 mmol/L CaCl_2_ with one mmol/L AlCl_3_⋅ 6H_2_O for 0, 4, 8, and 24 h. The CK group refers to the mock-treated control sampled at 24 h, which was collected at the same time point as the Al-treated samples but without Al exposure. Each treatment included three replicates. The sampling sites were divided into root and shoot, with two cm root tip tissue serving as the root samples, and leaf tissue serving as the shoot samples.

After collection, samples were immediately placed in a sterile mortar and frozen with liquid nitrogen, then ground into a fine powder using a sterile grinding rod. The powdered samples were transferred into RNase-free 1.5 mL centrifuge tubes, filling each tube to approximately one-third of its volume, and immediately returned to liquid nitrogen to prevent thawing. Samples were subjected to treatments initiated at different time points but were collected simultaneously to ensure comparability; all samples were stored at −80 °C for RNA extraction.

### Reverse transcription-quantitative polymerase chain reaction analysis

The reverse transcription-quantitative polymerase chain reaction (RT-qPCR) analysis was not performed using the same RNA (cDNA) samples as those used for Illumina sequencing. Independent biological samples were used for RT-qPCR validation to ensure the robustness and reproducibility of the expression patterns observed in the transcriptome data. Six *CpWRKY* genes (*CpWRKY013*, *CpWRKY049*, *CpWRKY076*, *CpWRKY114*, *CpWRKY126*, and *CpWRKY162*) were randomly selected from 48 DEGs in elephant grass for RT-qPCR. Total RNA was extracted from root and shoot tissues using TRIzol reagent (Sangon Biotech Co., Ltd., Shanghai, China). Residual genomic DNA was removed prior to reverse transcription. For each reaction, 1,000 ng of RNA (adjusted based on the initial concentration) was mixed with 3 µL of SX gDNA Digester Mix and of RNase-free ddH_2_O up to 15 µL, gently pipetted, and incubated at 42 °C for 15 min in a PCR instrument. The reverse transcription reaction (20 µL total volume) was then prepared by adding 5 µL of 4 × III-MLvRT Mix to 15 µL of the DNA-depleted RNA. Reverse transcription was performed on a PCR system under the following conditions: 25 °C for 5 min, 55 °C for 15 min, and 85 °C for 5 min. The resulting cDNA was diluted with RNase-free DEPC water to a final concentration of 100 ng/µL.

Specific primers for the target genes were designed with lengths of 18–25 bp, GC content of 40–60%, amplicon sizes of 100–200 bp, melting temperatures (Tm) of 58–62 °C with ≤ 1 °C difference between primer pairs, and spanning exon–exon junctions (primer sequences are listed in Table S2). *CpEF1*α (*GWHTAORA004699*) served as the reference gene. For RT-qPCR, a 10 µL reaction mixture was prepared using 2 ×SG Fast qPCR Premix (5 µL), forward and reverse primers (0.2 µL each), cDNA template (1 µL), and ddH_2_O (3.6 µL). Amplification was performed on a LightCycler 480 II system (Roche Diagnostics, Basel, Switzerland) with the following program: 95 °C pre-denaturation for 30 s; 40 cycles of 95 °C denaturation for 10 s and 60 °C annealing/extension for 30 s; followed by melting curve analysis from 65–95 °C. Each sample was analyzed in three biological replicates. Cq values were automatically determined by the second derivative maximum method using LightCycler 480 II system. Relative gene expression levels were calculated using the 2^^−^ΔΔCt^ method normalized to *CpEF1*α.

### Illumina sequencing and sequence assembly

RNA integrity of the root and shoot samples was evaluated using the RNA Nano 6000 Assay Kit in combination with the Bioanalyzer 2100 system (Agilent Technologies, CA, USA). First-strand cDNA was synthesized with random hexamer primers and M-MuLV Reverse Transcriptase (RNase H-deficient), followed by second-strand synthesis using DNA Polymerase I and RNase H. The resulting overhangs were converted to blunt ends, and 3′ adenylation enabled ligation of adaptors containing hairpin loops. Libraries were enriched by PCR using high-fidelity DNA polymerase and indexed primers, purified with magnetic beads, and their quality was confirmed on the Bioanalyzer 2100. Indexed libraries were clustered on a cBot Cluster Generation System employing the TruSeq PE Cluster Kit v3-cBot-HS and subsequently sequenced on an Illumina NovaSeq platform to generate 150-bp paired reads.

## Results

### Comprehensive identification and analysis of the WRKY TF family in elephant grass

**Figure 1:**
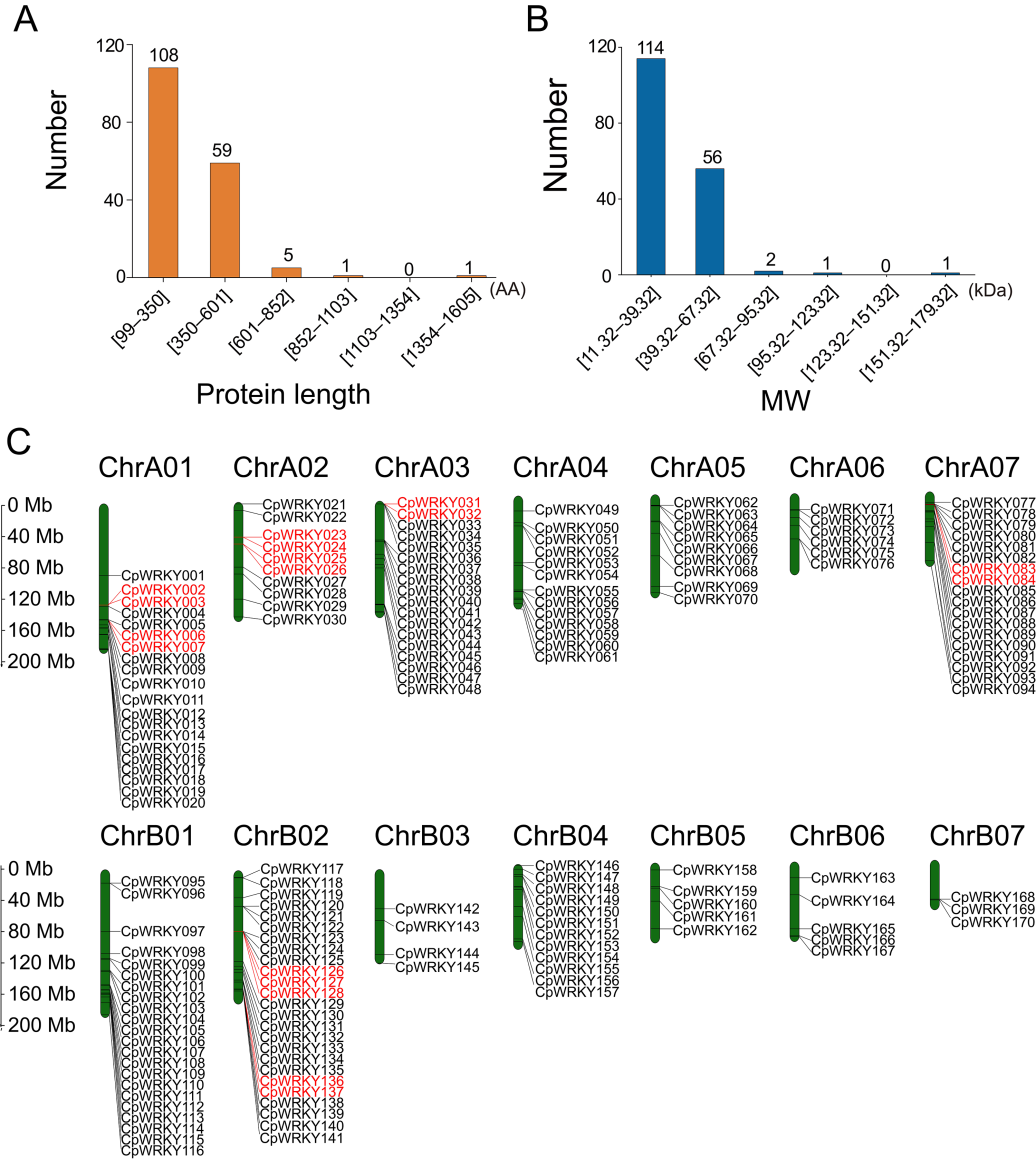
The physicochemical property analysis and gene mapping of CpWRKY members in elephant grass. (A) Distribution of protein counts across distinct amino acid (AA) length intervals. (B) Molecular weight (kDa) distribution of CpWRKY proteins. (C) The positions of *CpWRKY* genes on chromosomes of elephant grass. The gene names highlighted in red indicate tandem duplication events.

In elephant grass, 174 WRKY TFs were identified and designated CpWRKY001 to CpWRKY174 according to their chromosomal order (Table S3). The number of amino acids ranged from 99 (CpWRKY006) to 1,602 (CpWRKY028) (Fig. 1A, Table S4), the theoretical isoelectric points (pI) spanned from 4.58 (CpWRKY120) to 11.38 (CpWRKY025), and the molecular weights varied from 11.32 kDa (CpWRKY114) to 178.27 kDa (CpWRKY028) (Fig. 1B, Table S4). Protein stability analysis indicated that among these 174 CpWRKY proteins, six WRKY proteins (CpWRKY036, CpWRKY069, CpWRKY120, CpWRKY122, CpWRKY158, CpWRKY165) were stable proteins, while the remaining 168 CpWRKY TFs were all unstable proteins (Table S4).

### Chromosomal location of WRKY TFs in elephant grass

Chromosomal localization revealed that 170 of 174 CpWRKY TFs were unevenly distributed across 14 chromosomes (Fig. 1C), while the remaining four CpWRKY TFs (CpWRKY171, CpWRKY172, CpWRKY173, and CpWRKY174) were located in unassembled contigs (Fig. S1). Among these 14 chromosomes, chromosome ChrB02 had the largest number of CpWRKY TFs (25), and ChrB07 had the fewest (three). The remaining chromosomes showed varying numbers of CpWRKY TFs, with ChrA01 containing 20, ChrA02 (10), ChrA03 and ChrA07 with 18, ChrA04 (13), ChrA05 (9), ChrA06 (6), ChrB01 (22), ChrB03 (4), ChrB04 (12), ChrB05 and ChrB06 with five genes.

### Classification and phylogenetic analysis of CpWRKY TFs

**Figure 2:**
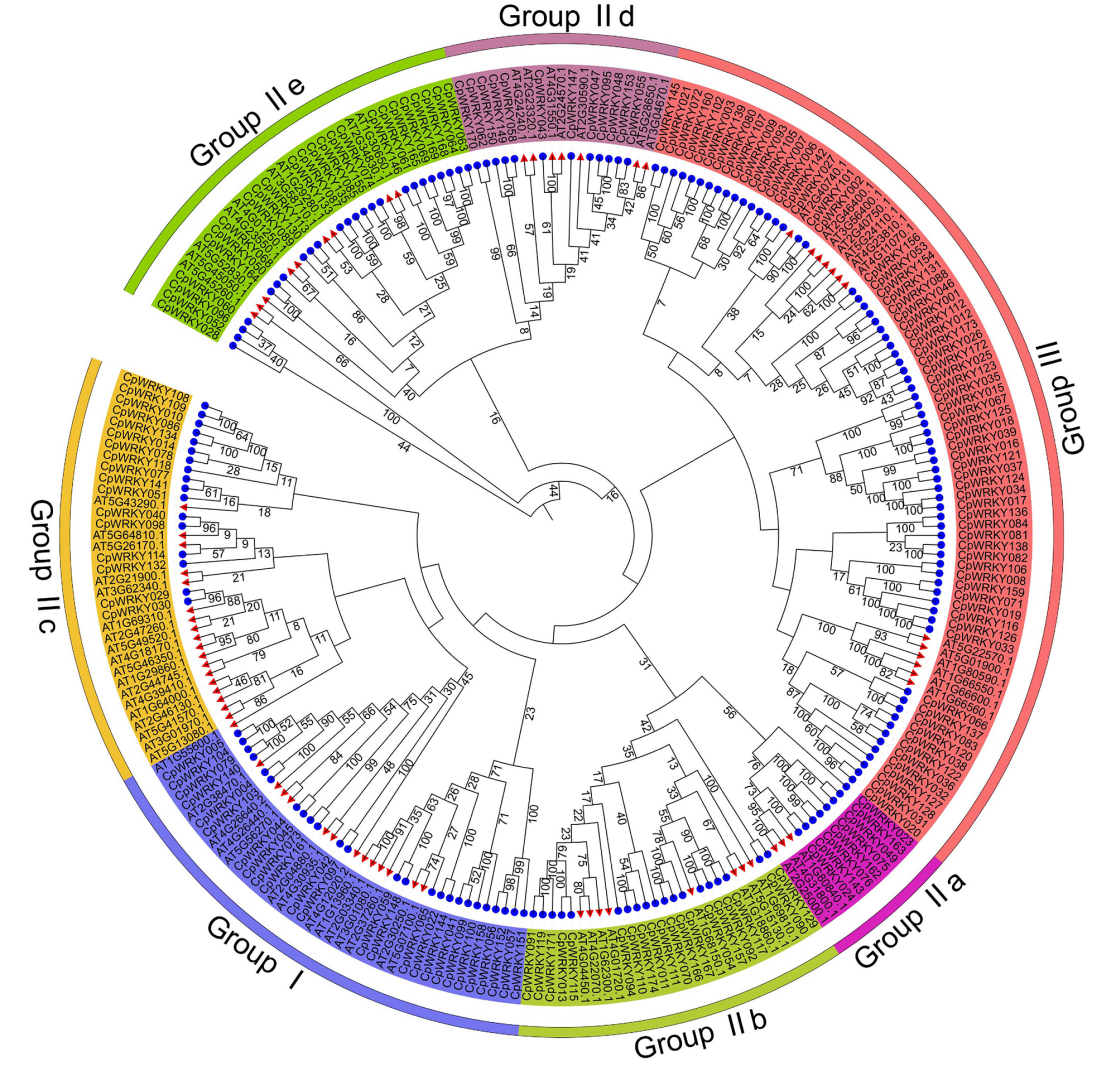
Phylogenetic tree of CpWRKY and AtWRKY proteins. The outer Roman numerals represent different subfamily classifications, with red triangles indicating Arabidopsis WRKY proteins and blue circles representing elephant grass WRKY proteins. The numbers at branch nodes or alongside branches in a phylogenetic tree usually represent bootstrap values (self-support rates).

To explore the evolutionary relationships of CpWRKY TFs, a phylogenetic tree was generated by combining 174 CpWRKY proteins from elephant grass with 72 WRKY proteins from Arabidopsis (Fig. 2). Based on the sequence similarity, WRKY TFs were divided into seven groups containing Group I, Group IIa, Group IIb, Group IIc, Group IId, Group IIe, and Group III, with the number of members per subgroup ranging from 11 (Group IIa) to 82 (Group III). Twenty-five CpWRKY TFs and 14 AtWRKY TFs belonged to Group I, eight CpWRKY TFs and three AtWRKY TFs were part of Group IIa, 19 CpWRKY TFs and eight AtWRKY TFs were assigned to Group IIb, 17 CpWRKY TFs and 18 AtWRKY TFs were grouped into Group IIc, 12 CpWRKY TFs and seven AtWRKY TFs were sections of Group IId, 24 CpWRKY TFs and nine AtWRKY TFs of Group IIe, 69 CpWRKY TFs and 13 AtWRKY TFs were sections of Group III.

### Motif analysis, domain mapping, and gene structure of CpWRKYs

**Figure 3:**
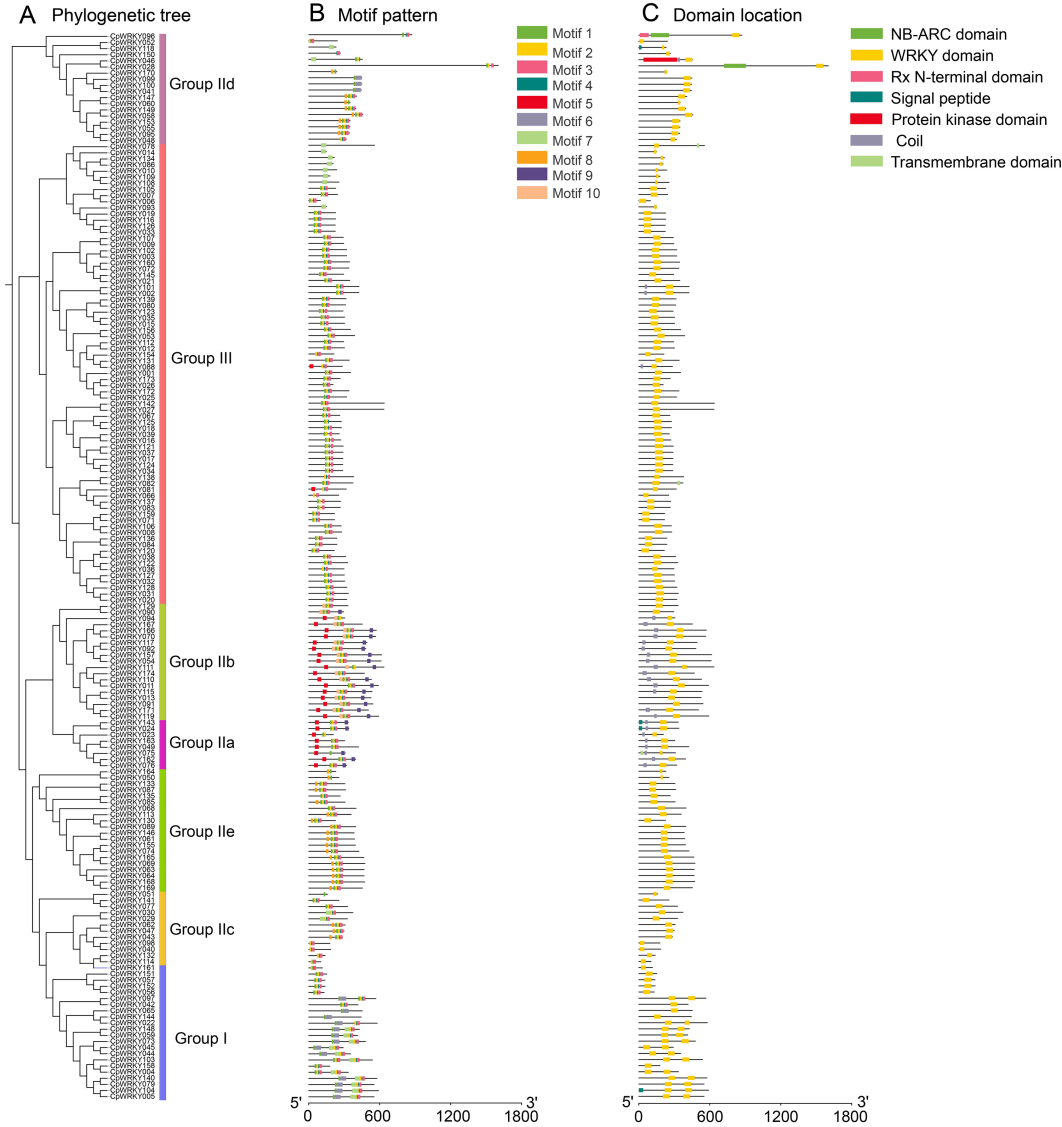
Phylogenetic relationships, motif analysis and domains location of CpWRKY TFs. (A) Phylogenetic relationships of CpWRKY proteins. (B) Distribution of 10 conserved motifs (numbered 1–10) in CpWRKY proteins, represented by distinct colored boxes. (C) Locations of WRKY domains in CpWRKY proteins.

Phylogenetic tree analysis showed that Group III contains the most CpWRKY members (69), while Group IIa contains the fewest members (eight) (Fig. 3A). Motif analysis showed that a total of 10 motifs were identified in 174 CpWRKY protein sequences (Fig. 3B). The structural features of motifs 1–10 are listed in Table S5, and the positional distribution of motifs 1–10 among the 174 CpWRKY proteins is shown in Table S6. Motif 9 is specific to Group II. Motif 6 exists only in Group IId and Group I. Among all WRKY TFs, the number of motifs contained varies, with a minimum of one motif and a maximum of seven motifs. The results of domain location analysis indicated that the WRKY domain is present in all WRKY TFs. Most members of Group I contain only the WRKY domain, whereas CpWRKY104 is unique in possessing an additional signal peptide domain. The WRKY proteins in Groups IId and IIa have a greater variety of domain types, among them, CpWRKY096, CpWRKY046, CpWRKY143, CpWRKY024, and CpWRKY075 contain three domains (Fig. 3C). Members of Groups IIc and IIe only contain one domain. The sequence positions of the major domains in the 174 CpWRKY members are provided in Table S7. Through integrated motif and domain analysis, it was found that motifs 1, 2, and 4 completely overlap with the WRKY domain, motifs 3, 6, and 7 partly overlap with the WRKY domain, while the remaining motifs show no overlap (Tables S5, S6, and S7). Gene structure and evolutionary tree analysis showed that all *CpWRKY* genes contain exons ranging in number from one to nine. *CpWRKY171* has the largest number of exons (nine) (Fig. S2). Group IIc and Group IIa have the smallest number of exons, with most containing only one to four, except for *CpWRKY047* in Group IIc, which contains six. Group IIb has the largest number of exons, with the fewest members having two and the most having nine.

### Secondary structure and 3D models of CpWRKY proteins

Three representative CpWRKY proteins from each of the seven phylogenetic groups were selected for analysis of their secondary structures and 3D conformations to explore structure–function relationships. The secondary structures of these 21 CpWRKY proteins were predicted, revealed that random coils accounted for the largest proportion (38.89–88.41%), followed by α-helices (4.71–52.25%), extended strands (4.28–13.68%), and β-turns (0.00–4.25%) (Table S8). Among the 21 modeled CpWRKY proteins, 19 had GMQE scores greater than 0.5, while the other two had scores of 0.49 (CpWRKY028, CpWRKY030), indicating that the models were of high structural reliability (Fig. S3).

### Synteny and evolutionary pressure analysis

**Figure 4:**
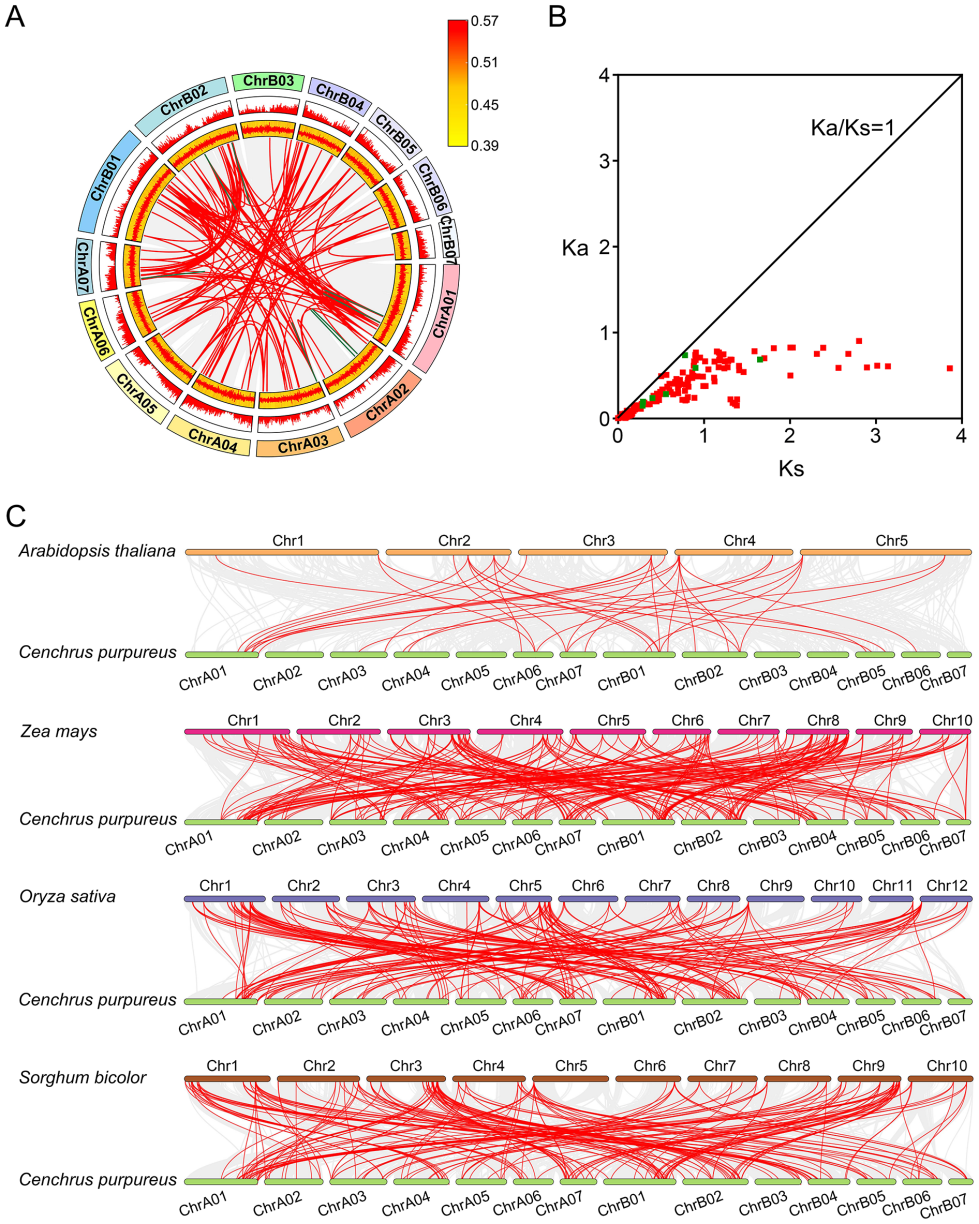
Synteny analysis of *CpWRKY* genes. (A) Intraspecific collinearity analysis within elephant grass: Gray lines represent all collinear relationships in elephant grass, red highlighted lines indicate the segmental duplication of *CpWRKY* genes in elephant grass, and green lines represent the occurrence of tandem duplication events. Gene density is represented by yellow boxes, and the GC content of *CpWRKY* genes is shown in white boxes. (B) Evolutionary pressure analysis of collinear *CpWRKY* gene pairs: red squares indicate Ka/Ks values for segmentally duplicated gene pairs, green squares for tandemly duplicated pairs, and the black line represents Ka/Ks = 1. (C) Inter-species synteny analysis: Gray lines show collinear blocks between elephant grass and four reference species, and red lines indicate syntenic gene pairs involving *CpWRKY* genes.

Among the 174 *CpWRKY* genes, 178 collinear gene pairs were detected (Fig. 4A), including 169 segmental duplication pairs and nine tandem duplication pairs (Table S9). Tandem duplication events of nine gene pairs occurred on chromosomes ChrA01, ChrA02, ChrA03, ChrA07, and ChrB02, respectively (Table S10). All examined gene pairs exhibited Ka/Ks ratios below one (Fig. 4B). Synteny analysis across species demonstrated significant collinearity between elephant grass and four reference species, with 36, 270, 218, and 214 gene pairs identified in Arabidopsis, maize, sorghum, and rice, respectively. Twenty-seven *CpWRKY* genes maintained syntenic links with all four species, whereas 116 *CpWRKY* genes showed synteny with three Poaceae species (maize, rice, and sorghum) (Fig. 4C).

### Expression analysis profiles of the *CpWRKY* gene family in different tissues

Within the 174 *CpWRKY* genes, four genes (*CpWRKY011*, *CpWRKY066*, *CpWRKY089, and CpWRKY137*) showed no detectable expression (TPM = 0) across all tested tissues, while the remaining 170 genes were expressed in at least one tissue. By applying dual thresholds—a fold change of ≥ 2 and a TPM value greater than 10, we defined our tissue-specific highly expressed genes (Table S11). The results revealed that two genes were tissue-specific highly expressed in roots, with TPM values ranging from 10.52 (*CpWRKY164*) to 15.32 (*CpWRKY098*) and fold changes ranging from 2.07 (*CpWRKY164*) to 2.15 (*CpWRKY098*). Eighteen genes were specifically highly expressed in stems, with TPM values ranging from 12.85 (*CpWRKY079*) to 188.24 (*CpWRKY114*), and fold changes ranging from 2.43 (*CpWRKY093*) to 5460.00 (*CpWRKY123*). Four genes were specifically highly expressed in stem tips, with TPM values ranging from 10.95 (*CpWRKY074*) to 308.74 (*CpWRKY118*) and fold changes ranging from 2.07 (*CpWRKY149*) to 24.37 (*CpWRKY155*). Eighteen genes were specifically highly expressed in leaves, with TPM values ranging from 10.33 (*CpWRKY134*) to 135.45 (*CpWRKY114*) and fold changes ranging from 2.41 (*CpWRKY114*) to 288.77 (*CpWRKY092*). Two genes were specifically highly expressed in flowers, with TPM values ranging of 10.80 (*CpWRKY040*) and 12.94 (*CpWRKY134*), and fold changes of 3.91 (*CpWRKY134*) and 6.24 (*CpWRKY040*).

**Figure 5:**
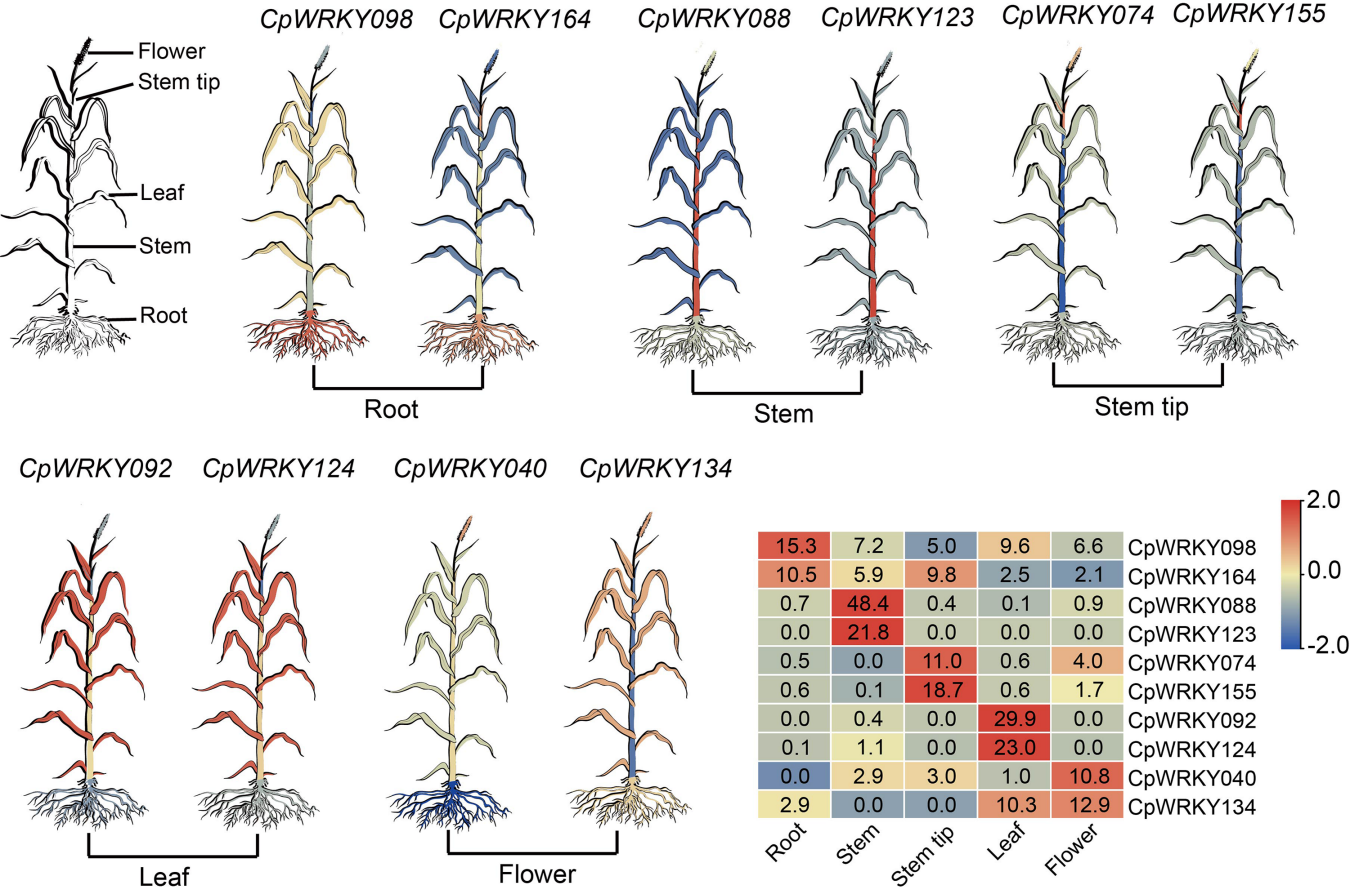
Expression pattern diagram of *CpWRKY* genes in five elephant grass tissues, alongside a heatmap illustrating the expression levels of the ten selected genes.

In each tissue, the two most highly expressed genes were chosen for visualization: *CpWRKY098* and *CpWRKY164* (root), *CpWRKY088* and *CpWRKY123* (stem), *CpWRKY074* and *CpWRKY155* (stem tip), *CpWRKY092* and *CpWRKY124* (leaf), *CpWRKY040* and *CpWRKY134* (flower) (Fig. 5).

### Expression analysis of *CpWRKY* genes under Al stress

**Figure 6:**
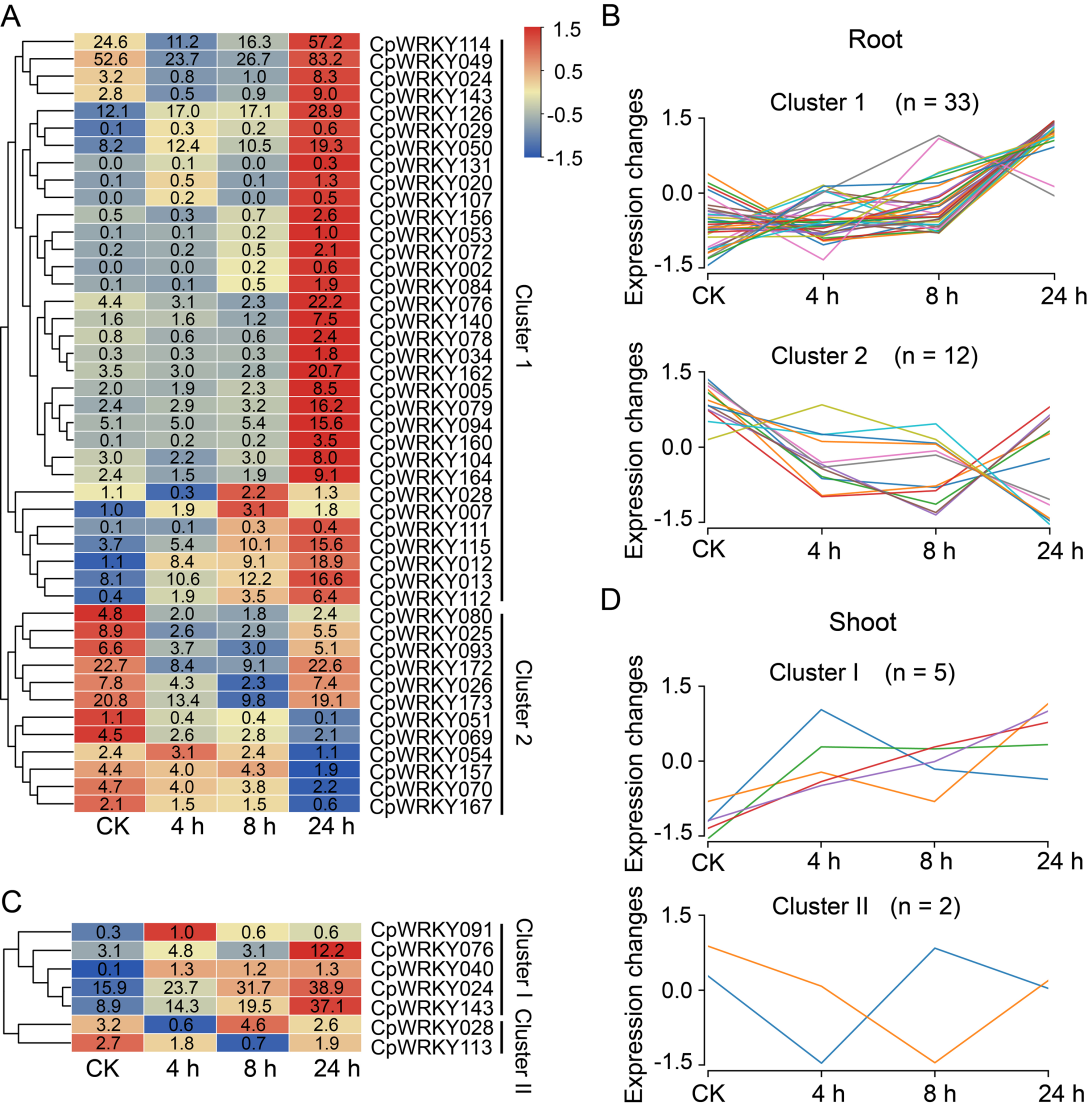
Expression analysis of *CpWRKY* genes under Al treatment at different time points. (A) Heatmap showing the DEGs in roots compared with the control group. Cluster 1 represents the upregulated genes after 24 h Al treatment compared with the CK group, and Cluster 2 represents the downregulated genes after 24 h Al treatment compared with the CK group. (B) Line chart showing the expression trends of Cluster 1 and Cluster 2 in roots. (C) Heatmap showing the DEGs in shoots compared with the control group. Cluster I represents the upregulated genes after 24 h Al treatment compared with the CK group, and Cluster II represents the downregulated genes after 24 h Al treatment compared with the CK group. (D) Line chart showing the expression trends of Cluster I and Cluster II in shoots.

During transcriptome data analysis, genes with —log_2_FoldChange— ≥ 1 and Padj < 0.05 in at least two treatment time points were considered as DEGs. In roots, 45 *CpWRKY* genes were identified as significantly differentially expressed under the short-term Al stress treatment (Fig. 6A), consisting of 33 genes with increased expression and 12 with decreased expression (Fig. 6B). In shoots, seven *CpWRKY* genes showed differential expression (Fig. 6C), of which five exhibited increased expression and two exhibited decreased expression (Fig. 6D). As four genes (*CpWRKY024*, *CpWRKY028*, *CpWRKY076*, and *CpWRKY143*) were differentially expressed in both roots and shoots, a total of 48 DEGs were identified in elephant grass under short-term Al treatment conditions. In roots, genes such as *CpWRKY020, CpWRKY079*, and *CpWRKY115* showed significant upregulation after 24 h of Al treatment, while genes like *CpWRKY157, CpWRKY070*, and *CpWRKY167* were significantly downregulated after 24 h of Al treatment. In shoots, genes including *CpWRKY024* and *CpWRKY143* remained highly expressed after 24 h Al treatment, and *CpWRKY028* and *CpWRKY113* reached their lowest values at 4 h and 8 h of Al treatment, respectively. Genes whose expression levels showed no substantial changes at 0, 4, 8, and 24 h under Al treatment were considered not to be involved in the short-term Al stress response.

### Promoter *cis*-acting element analysis of *CpWRKY* genes

**Figure 7:**
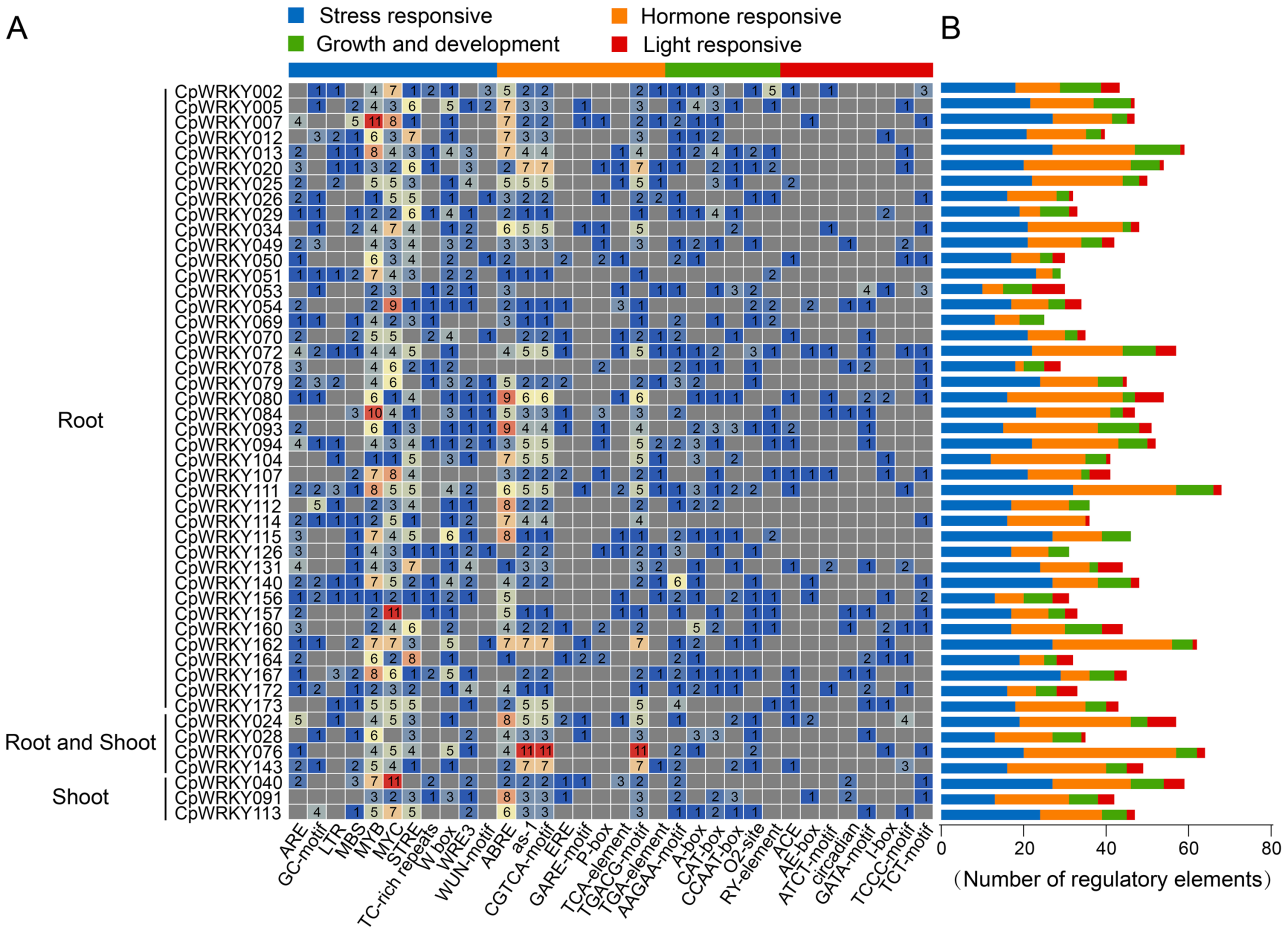
*Cis*-regulatory element analysis of *CpWRKY* gene promoter regions in elephant grass. (A) The number of each *cis*-regulatory element in the 2,000 bp upstream promoter regions of *CpWRKY* genes. (B) The number of cis-elements related to plant stress, hormones, growth, and light responses in the promoter regions of *CpWRKY* genes.

By identifying and analyzing *cis*-acting elements in the promoter regions of genes, we can explore the potential roles of these elements in plant responses to biotic and abiotic stresses, providing valuable insights into the mechanisms of stress regulation (Fig. 7). In this study, *cis*-acting regulatory elements of 48 DEGs under 24-h time-course Al treatment were identified. These regulatory elements were classified into four types: stress response, hormone response, plant growth and development, and light response elements. Among these 48 *CpWRKY* DEGs, *CpWRKY111* possessed the most regulatory elements (68), whereas *CpWRKY068* contained the fewest (25). Regarding stress-responsive elements, *CpWRKY053* had the fewest (10), and *CpWRKY111* had the most (32). For hormone-responsive elements, *CpWRKY078* had the fewest (two), while *CpWRKY076* had the most (37). In terms of growth and development-related elements, *CpWRKY114* had none, whereas *CpWRKY013* had the most (11). For light-responsive elements, *CpWRKY051*, *CpWRKY126*, *CpWRKY112*, *CpWRKY069*, and *CpWRKY115* lacked light-responsive elements, while *CpWRKY053* contained the most (eight).

### RT-qPCR analysis

**Figure 8:**
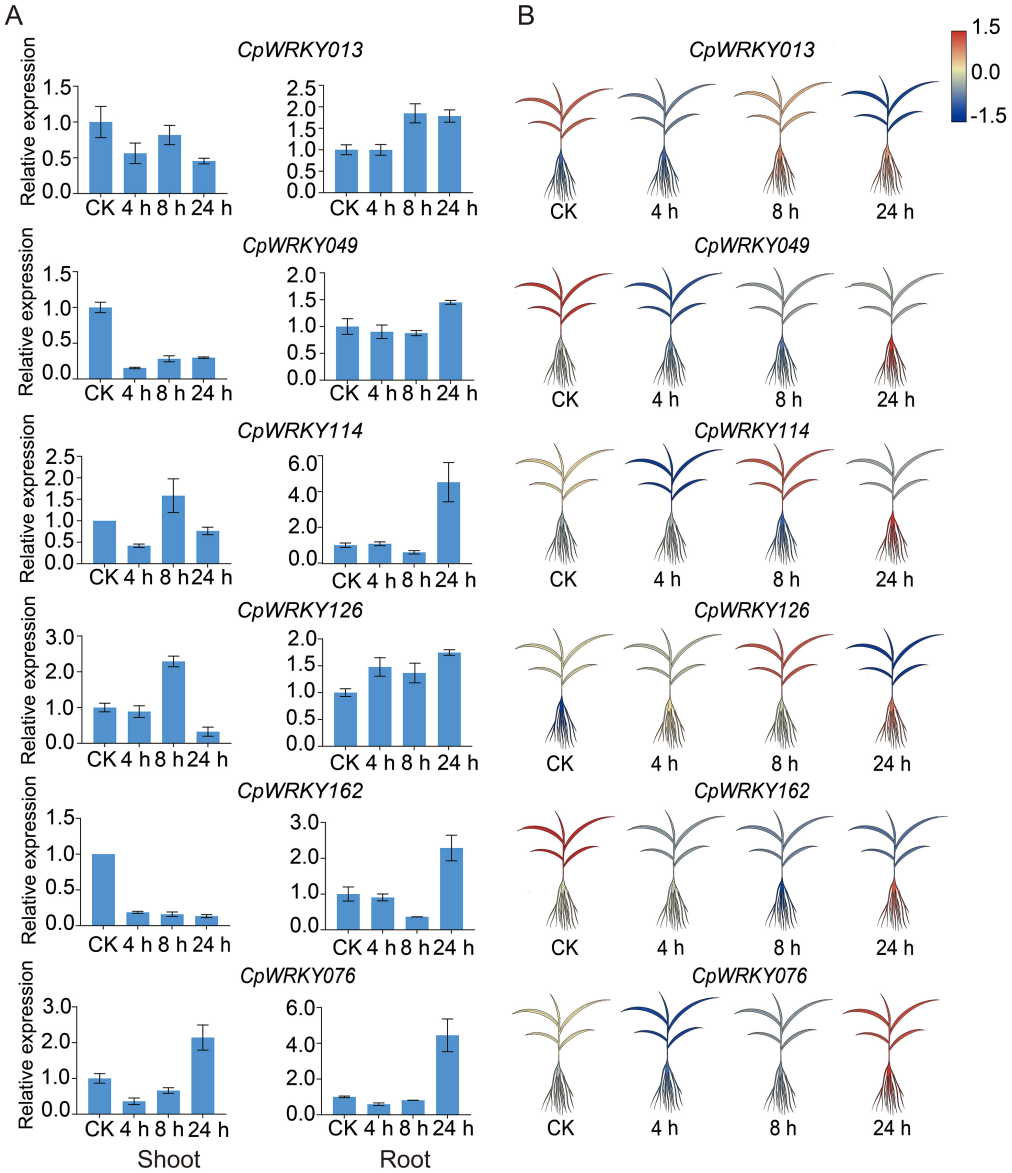
RT-qPCR of six *Cp WRKY* genes. (A) Expression of six selected *CpWRKY* genes at four Al treatment time points. (B) Schematic representation of RT-qPCR results in elephant grass.

Six genes (*CpWRKY013*, *CpWRKY049*, *CpWRKY076*, *CpWRKY114*, *CpWRKY126,* and *CpWRKY162*) were randomly selected from the 48 DEGs mentioned above for further RT-qPCR analysis under the 24-h time-course Al stress treatment (Figs. 8A, 8B). Among the six genes, five (*CpWRKY013*, *CpWRKY049*, *CpWRKY114*, *CpWRKY126,* and *CpWRKY162*) showed downregulation in shoots but upregulation in roots, while one gene (*CpWRKY076*) was upregulated in both tissues. In roots, *CpWRKY076* showed the lowest expression after 4 h Al treatment, while *CpWRKY049*, *CpWRKY114*, and *CpWRKY162* reached their lowest levels after 8 h treatment. In contrast, *CpWRKY049*, *CpWRKY076*, *CpWRKY114*, *CpWRKY126*, and *CpWRKY162* all reached their peak expression levels after 24 h Al treatment, *CpWRKY013* peaked after 8 h treatment. In shoots, *CpWRKY013*, *CpWRKY126*, and *CpWRKY162* reached their lowest levels after 24 h treatment, while *CpWRKY049*, *CpWRKY076*, and *CpWRKY114* reached their lowest expression levels after 4 h Al treatment. Conversely, *CpWRKY076* peaked after 24 h treatment, while *CpWRKY114* and *CpWRKY126* peaked after 8 h Al treatment.

Additionally, the relative expression of *CpWRKY114* and *CpWRKY076* in roots increased more than fourfold after 24 h of Al treatment, indicating that they may participate in the late transcriptional response to short-term Al stress and represent key candidate genes for elucidating Al-responsive regulatory mechanisms (Fig. 8A).

### Protein-DNA interaction, protein-protein interaction analysis of CpWRKY TFs

**Figure 9:**
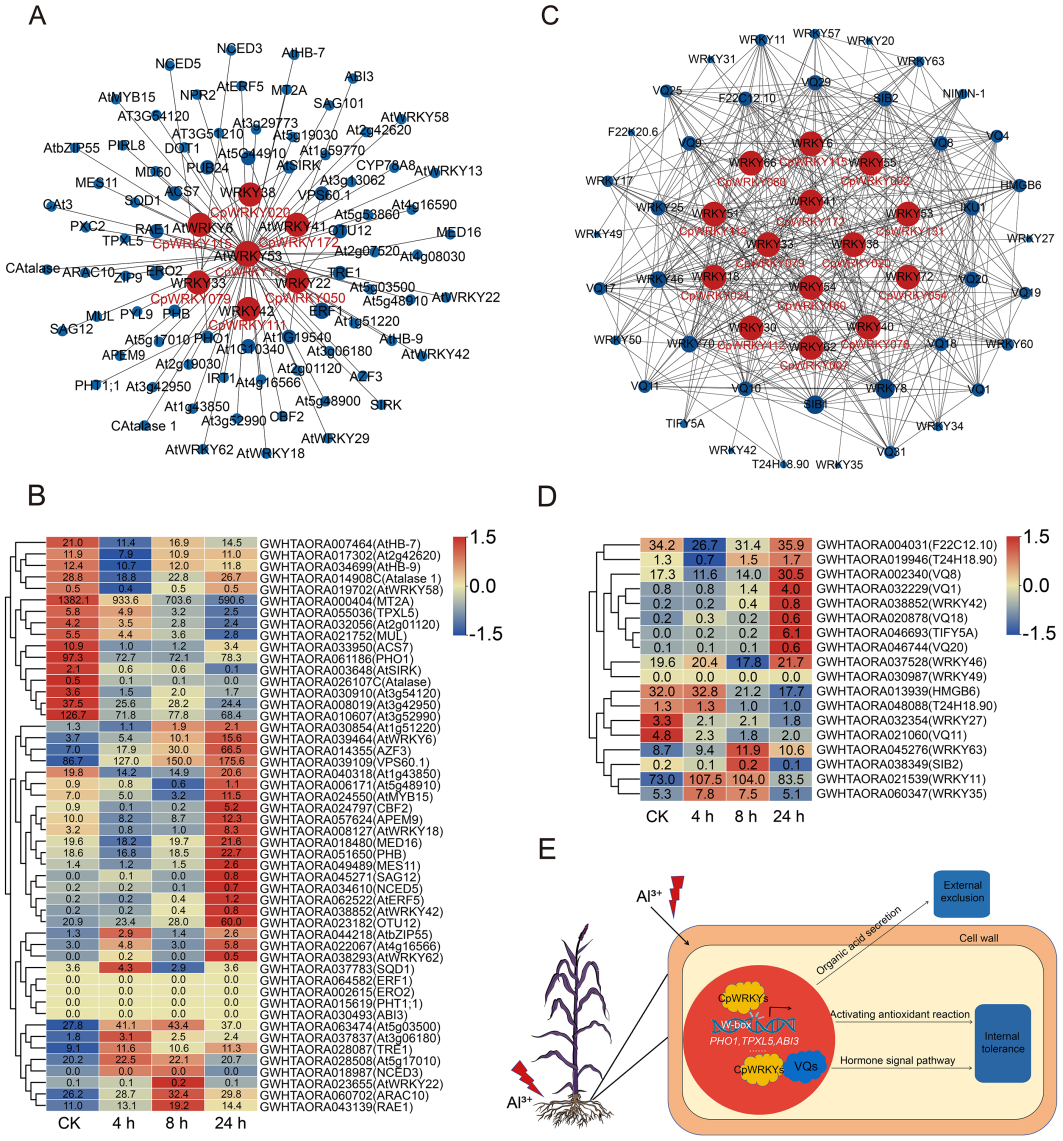
PDI, PPI network diagram, and expression analysis of interacting genes. (A) Predicted PDI network of *CpWRKY* genes based on their Arabidopsis orthologs. (B) Expression levels of the target genes in roots regulated by seven CpWRKY TFs. (C) Predicted protein interaction network based on orthologs of *CpWRKY* genes from Arabidopsis. (D) Expression analysis of the genes homologous to Arabidopsis in the protein-protein interaction network of elephant grass. (E) A diagram of the Al regulatory mechanisms potentially mediated by CpWRKY TFs in elephant grass. (B) and (D) display gene expression levels as Z-scores of log_2_(FPKM), calculated from three biological replicates. Blue and red indicate low and high expression, with heatmap values reflecting the original FPKM data.

Among 48 *CpWRKY* DEGs mentioned above, seven homologous proteins—CpWRKY020 (ortholog of AtWRKY38), CpWRKY115 (AtWRKY6), CpWRKY172 (AtWRKY41), CpWRKY025 (AtWRKY53), CpWRKY079 (AtWRKY33), CpWRKY111 (AtWRKY42), and CpWRKY050 (AtWRKY22)—were predicted to target 82 downstream Arabidopsis genes, but only 50 of them could be matched with elephant grass genes (Fig. 9A, Table S12). Among these 50 elephant grass genes, 16 were differentially expressed under Al treatment conditions, with 10 genes significantly upregulated and six genes significantly downregulated (Fig. 9B). Prediction of W-box elements in these 50 potential downstream regulatory genes revealed that 43 of them contain W-box elements (Table S12). GO analyses of the downstream regulatory genes showed that a total of 27 terms were significantly enriched, among which “sequence-specific DNA binding” was the most prominent, followed by “oxidoreductase activity” and “peroxidase activity” (Fig. S4A, Table S13). The results of KEGG analysis indicated that the downstream genes were significantly enriched in 15 pathways, with the “MAPK signaling pathway” being the most significant, followed by the “glyoxylate and dicarboxylate metabolism” and “peroxisome” pathways (Fig. S4B, Table S14).

Through PPI network analysis, several interaction partners were identified for the candidate CpWRKY TFs. Fourteen out of 48 candidate TFs were predicted to interact with 53 target proteins (Fig. 9C; Table S15). Not only do WRKY proteins exhibit strong interactions with each other, but they also show significant interactions with other proteins, such as VQ proteins. Further expression analysis of the homologous elephant grass genes for these 53 potential interacting proteins revealed that 18 Arabidopsis genes could pair with elephant grass. Among them, 10 genes peaked at 24 h of Al treatment, four gradually declined under Al stress, and four reached maximum expression at 4 and 8 h (Fig. 9D). *CpWRKY114* and *CpWRKY076* were strongly induced by 24 h Al treatment in roots, with *CpWRKY076* also upregulated in leaves, and both were predicted to participate in the PPI network, suggesting potential roles in Al stress response (Figs. 8A, 8B, and 9B). In addition, by combining PDI and PPI networks, we found that CpWRKY131, CpWRKY172, CpWRKY079, and CpWRKY115 consistently appeared in both networks and CpWRKY131 showed multiple downstream interactions, suggesting a potentially central regulatory role.

## Discussion

In regions with acidic Al soils, elephant grass demonstrates vigorous root growth, accumulates low levels of Al^3+^, and maintains high photosynthetic efficiency (Yan et al., 2021). This suggests that elephant grass has unique Al-tolerance mechanisms, making it as a valuable model for understanding the molecular basis of plant responses to Al stress. This study lays a foundation for understanding the roles of CpWRKY TFs in Al response and provides potential gene resources for improving tolerance in sensitive species.

We identified 174 *WRKY* genes in elephant grass, whereas Mao et al. (2024) reported 176. All 174 genes identified in our study were included in their dataset, with only *CpA0202180.1* and *CpB0301231.1* unique to their results. This discrepancy arises from distinct gene identification pipelines: we performed BLAST searches against WRKY sequences of Arabidopsis, rice and maize, followed by HMM, InterPro and CDD validation, whereas Mao et al. (2024) only used HMM-based and CDD-based screening. In addition, Mao et al. (2024) analyzed cold responses in the elephant grass cultivar ‘CN002’, whereas our study investigated aluminum stress responses in the cultivar ‘Purple’. Variations in cultivar and stress treatment are likely to induce distinct genetic and physiological responses. The CpWRKY proteins exhibited substantial variation in molecular weight and isoelectric point, which may be related to their functional diversity under different environmental conditions (Wu et al., 2025). Subcellular localization prediction revealed that most CpWRKY proteins (90.8%) are located in the nucleus, consistent with their typical roles as TFs that function by entering the nucleus to bind promoter regions and regulate gene expression (Wang et al., 2011). Approximately 10% of CpWRKY proteins are localized to extranuclear compartments such as the cytoplasm, mitochondria, and chloroplasts. Previous studies have indicated that TFs localized outside the nucleus can be activated by specific biotic or abiotic stress signals and may translocate between organelles to exert their regulatory functions (Marathe, Grotewold & Otegui, 2024). These TFs might therefore have significant roles in stress signaling and adaptation. Phylogenetic analysis showed that the 174 CpWRKY proteins could be classified into three groups: I, II, and III. Group I contains 25 members, Group III has 69, and Group II, the largest group, includes 80 members. In Arabidopsis and poplar, Group I has the most WRKYs, while in rice, Group III is the largest (Xu et al., 2020). In elephant grass, the largest group is Group II, indicating that this group may have experienced more extensive gene duplication during evolution.

Multiple sequence alignment revealed that 118 CpWRKY proteins possess a highly conserved heptapeptide core sequence (WRKYGQK) (Table S3). Among them, 11 CpWRKYs contain two “WRKYGQK” conserved motifs, and all belong to Group I, a feature also found in Arabidopsis (Eulgem et al., 2000) and maize (Wei et al., 2012). It has been shown that the “WRKYGQK” motif interacts with W-box elements to control target gene expression (Suttipanta et al., 2011). Six types of heptapeptide sequence variants (WKKYGQK, WRKYGEK, WRKYGKK, WRKYRQK, WRKGLEF, and WLKYGEK) were identified in Groups I, IIb, IIc, IIe, and III. Variations in the WRKYGQK domain may affect its binding specificity and develop a new regulatory mechanism. Some CpWRKY proteins lack the canonical WRKYGQK motif or the zinc-finger structure, representing atypical WRKY variants generated through sequence divergence after gene duplication, and these WRKY proteins may instead function through alternative regulatory mechanisms in stress responses (Table S3). Motifs 1, 2, and 4 completely overlap with the WRKY domain, confirming their participation in DNA binding and controlling transcription. Motifs 3, 6, and 7 partly overlap with the WRKY domain, while the remaining motifs show no overlap, suggesting functional diversification among CpWRKY proteins. A similar distribution pattern, characterized by both overlaps and distinctions between motifs and domains, has also been reported for the elephant grass MYB gene family (Wang et al., 2025). This finding indicates that conserved motifs represent key functional domains, while motif variations contribute to the functional divergence of TFs.

Gene duplication is essential for species evolution, genome expansion, and gene family diversification (Zhang et al., 2023). In this study, extensive synteny was observed between *WRKY* genes of elephant grass and those of maize, rice, and sorghum, whereas fewer syntenic relationships were detected with Arabidopsis, consistent with the evolutionary divergence between monocots and dicots (Mangelsen et al., 2008). This study identified 168 pairs of segmentally duplicated and nine pairs of tandemly duplicated *CpWRKY* genes in elephant grass, revealing that segmental duplication is the primary factor driving WRKY TF family expansion (Fig. 4). This pattern is highly consistent with the observations in watermelon (*Citrullus lanatus*) (Yang et al., 2018) and pineapple (*Ananas comosus*) (Xie et al., 2018), offering further confirmation of the major role played by segmental duplication in the evolution of plant TF families. A total of nine gene pairs exhibiting tandem duplication were detected in elephant grass, which is more than the number found in globe artichoke (*Cynara scolymus,* two pairs), carrot (*Daucus carota,* one pair), sunflower (*Helianthus annuus,* five pairs), and lettuce (*Lactuca sativa,* two pairs) (Guo et al., 2019). This difference may reflect the more complex genome duplications and rearrangements that occurred during the evolution of elephant grass.

WRKY TFs are involved in Al stress response in a range of plant species, such as Arabidopsis (Li et al., 2020), soybean (Shu et al., 2022), and rice (Li et al., 2018). In this study, 27.6% (48) of *CpWRKY* genes were identified as DEGs under Al stress conditions. Of these *CpWRKY* genes, 31 showed peak expression 24 h after Al treatment, such as *CpWRKY002* and *CpWRKY084* in roots, whereas *CpWRKY076* and *CpWRKY143* in shoots. In contrast, several *CpWRKY* genes achieved their expression peaks at the early stages of Al treatment (4 h or 8 h), such as *CpWRKY007*, *CpWRKY028*, and *CpWRKY054* in roots, as well as *CpWRKY028* and *CpWRKY091* in shoots. These results indicate that *CpWRKY* genes are regulated in both space and time under Al stress, with some acting early in signaling and others supporting long-term defense and metabolic balance. Interestingly, *CpWRKY002* in roots showed gradually increased expression under Al treatment, reaching its peak at 24 h, which is similar to its paralog, *SbWRKY22* (*Sobic.002G418500.1*) in sorghum that response to Al at late stage (Guan et al., 2023). This suggests that *CpWRKY002* is likely engaged in the response to Al stress across various species. In contrast, *CpWRKY084* exhibits the highest expression level at 24 h after Al treatment in roots, whereas its ortholog, *OsWRKY22* (*Os01g60490*), peaks at 3 h of Al treatment and then declines to a baseline level (Li et al., 2018). This suggests that the expression patterns of this gene in response to Al stress are diverse across different species. These findings suggest that WRKY members have retained certain conserved functions while also undergoing species-specific divergence in response to Al stress.

In the promoter regions of *CpWRKY* genes in elephant grass, a large number of *cis*-acting elements related to stress and hormone responses have been identified (Figs. 7A, 7B), including W-box, ABA-response elements (ABRE), SA-response elements (TCA elements) and JA-response elements (CGTCA motifs). ABA alleviates Al toxicity by enhancing antioxidant capacity and regulating the secretion of root organic acids (Salazar, Sanchez & Cruz, 2020), whereas JA and SA often enhance tolerance by inducing the expression of defense-related genes and promoting cell wall modification (Yang et al., 2017; Kwon et al., 2023). GmWRKY81 can bind to W-box elements to affect the transcriptional activity of 15 DEGs involved in Al stress, thereby enhancing Al tolerance in soybean (Shu et al., 2022). The widespread distribution of these elements suggests that CpWRKY TFs may function in multiple aspects of the Al response.

RT-qPCR analysis of the selected DEGs revealed substantial differences in their expression levels between shoots and roots. Under Al stress, *CpWRKY076* was consistently upregulated in both roots and shoots, suggesting that it participates in whole-plant, non-specific stress response pathways, such as oxidative stress signaling, hormone-mediated regulation, and basal defense cascades (Ma et al., 2025). These signals can be initiated directly in roots upon Al exposure and subsequently transmitted systemically to aerial tissues, resulting in coordinated induction of this gene in both organs. A similar pattern has been reported in Arabidopsis, where WAK1 showed parallel expression changes in roots and shoots after 9 h of Al treatment (Sivaguru et al., 2003). In contrast, *CpWRKY013, CpWRKY049*, and several other genes exhibited opposite expression trends between roots and shoots. This pattern reflects a division of labor between roots and shoots under stress: roots activate defense and stress-signaling pathways, while shoots reduce energy-consuming activities to save resources and support root adaptation, thereby improving whole-plant tolerance (Schmidt et al., 2014). Similar root-shoot divergence has been observed in other stresses, such as the opposite expression dynamics of GmHMA19 under Cd stress and GmHsfA1 under heat stress in soybean (Gao et al., 2017). Collectively, these tissue-dependent WRKY expression patterns highlight a functional partitioning of stress responses between organs, which contributes to the coordinated regulation of whole-plant Al tolerance. Notably, several genes exhibited differential expression across tissues and stress treatments. For example, *CpWRKY098* and *CpWRKY164* were highly expressed in roots in Milletdb but undetectable in our RNA-seq data. This discrepancy likely results from developmental stage differences: our RNA-seq data were from 30-day-old seedlings, whereas Milletdb’s tissue-specific profiles were derived from elephant grass plants at the flowering stage.

TFs regulate signal transduction and downstream functional gene expression by specifically binding to the promoter regions of target genes, thereby activating or repressing their transcription. In the PDI regulatory network, the homolog of CpWRKY172 in Arabidopsis, AtWRKY41 (*AT4G11070.1*), regulates the expression of abscisic acid-insensitive 3 (*ABI3*), which is enriched in “sequence-specific DNA-binding” GO term. ABI3 is a key TF that mediates ABA response, regulating plant responses to environmental stresses, as well as seed development and other physiological processes (Finkelstein, 1994). The orthologous gene of *ABI3* in elephant grass is *GWHTAORA030493*, which contains a W-box element (Table S12). The W-box is a specific sequence recognized by WRKY TFs and is crucial for plant gene regulation (Suttipanta et al., 2011). Thus, we speculate that CpWRKY172 may regulate the expression of *GWHTAORA030493* (*ABI3*) by binding to the W-box, thereby influencing the plant’s response to environmental stress. ABA-mediated stress regulation is conserved across species and stress types, and similar regulatory patterns have also been observed in elephant grass under cold stress (Hu et al., 2025). Furthermore, the ortholog of CpWRKY025, AtWRKY53, regulates *CAT1* and *CAT2*, both of which are enriched in GO term “antioxidant enzyme activity”. CAT1 and CAT2 catalyze the decomposition of H_2_O_2_ to remove reactive oxygen species (ROS) and maintain redox homeostasis in Arabidopsis (Bi et al., 2017). Therefore, it is hypothesized that CpWRKY025 may enhance stress resistance by regulating the ortholog genes *GWHTAORA014908* and *GWHTAORA026107* of *CAT1* and *CAT2* in elephant grass. AtWRKY53 also regulates the expression of the pyruvate kinase family gene *At3g52990*. The KEGG analysis found that *GWHTAORA010607*, ortholog of *At3g52990*, is enriched in the pyruvate metabolism pathway. Pyruvate can promote the synthesis and efflux of other organic acid metabolites (such as oxalic acid and malic acid) through interactions with them (Huber & Edwards, 1975). Organic acid exudation is a well-established Al tolerance mechanism in grasses such as barley and maize (Furukawa et al., 2007; Furuichi et al., 2010). Therefore, we propose that CpWRKY025 can also affect the secretion of organic acids by regulating *GWHTAORA010607*, thereby enhancing Al tolerance in elephant grass.

In plants, TFs interact with diverse proteins to coordinate signaling pathways and stress responses. Previous studies have revealed that several VQ proteins interact physically and functionally with WRKY TFs through the integrity of the VQ motif to regulate stress-related responses (Lai et al., 2011; Gayubas, Castillo & León, 2024). In the PPI regulatory network, CpWRKY079 is orthologous to Arabidopsis AtWRKY33, which has been shown to interact with two closely related VQ motif proteins, SIB1 and SIB2 in the nucleus. This interaction positively regulates plant defense responses against necrotrophic pathogens (Lai et al., 2011). The *SIB1* overexpression lines show significantly elevated transcription levels of ROS-related genes (Xie et al., 2010). Accordingly, CpWRKY079 in elephant grass is likely to interact with SIB1 and SIB2 to regulate ROS-scavenging responses under stress conditions. In addition, the orthologous of CpWRKY114, AtWRKY51, interacts with VQ1 and VQ10 in our predicted interaction network. VQ1 and VQ10 in Arabidopsis have been shown to promote photosynthesis and are upregulated under hypoxic, nitric oxide exposure, and oxidative stress conditions (Gayubas, Castillo & León, 2024). Overexpression of Poplar *VQ1* in Arabidopsis confers resistance to various biotic and abiotic stresses by mediating ABA and SA signaling pathways (Liu et al., 2023). This implies that CpWRKY114-VQ1/VQ10 module may be involved in Al responses *via* hormone signaling pathways in elephant grass.

Overall, both the transcriptional regulation and PPI mechanisms suggest that WRKY TFs in elephant grass may integrate multiple regulatory pathways, such as antioxidant defense system, organic acid secretion, and hormone signaling pathway, in response to Al stress (Fig. 9E). However, because these findings are based on bioinformatic predictions and functional inferences, further functional validation is necessary for these elephant grass genes. Despite these limitations, this study lays the groundwork for exploring Al tolerance mechanisms in elephant grass.

## Conclusion

In this study, 174 distinct CpWRKY TFs were identified and classified into seven subgroups. The exon-intron structures and domain characteristics of *CpWRKY* genes supported the validity of our classification results. Collinearity and evolutionary pressure analyses revealed that segmental duplication has played a major role in the expansion of the WRKY family. Expression profiles showed that a total of 48 *CpWRKY* genes exhibited differential expression at one or more time points under the treatment of one mmol L^−^^1^ AlCl_3_⋅ 6H_2_O, with 45 DEGs identified in the roots and seven in the shoots (including four commonly expressed in both tissues). Elements potentially involved in Al response, including W-box, ABRE, TCA, and CGTCA motifs, were also identified in the promoter regions of these 48 *CpWRKY* genes. PDI and PPI analyses indicated that CpWRKY TFs participate in antioxidant defense system, organic acid secretion, and hormone signaling pathway under Al stress. Future studies should validate the functions of identified *CpWRKY* genes (*CpWRKY076*, *CpWRKY114*, *CpWRKY131*, *etc.*) *via* transgenic overexpression and gene editing in an optimized elephant grass genetic transformation system, to clarify their regulatory roles. This will lay a solid foundation for dissecting the species’ stress response mechanisms and advancing its molecular breeding.

## Supplemental Information

10.7717/peerj.21299/supp-1 Supplemental Information 1 Genomic distribution of 4 * Cp WRKY* genes on unassembled contigs in elephant grass

10.7717/peerj.21299/supp-2 Supplemental Information 2 Exon-intron structures of * CpWRKY* genes, with exons depicted as light green boxes and introns as black lines

10.7717/peerj.21299/supp-3 Supplemental Information 3 The predicted 3D structural models of 21 CpWRKY proteins The model quality was evaluated using GMQE.

10.7717/peerj.21299/supp-4 Supplemental Information 4 GO and KEGG enrichment analyses of downstream elephant grass genes potentially regulated by CpWRKY TFs (A) GO enrichment analyses of downstream elephant grass genes potentially regulated by CpWRKY TFs. (B) KEGG enrichment analyses of downstream elephant grass genes potentially regulated by CpWRKY TFs.

10.7717/peerj.21299/supp-5 Supplemental Information 5 Locus ID information of WRKY TFs used as query sequences in BLASTp analysis

10.7717/peerj.21299/supp-6 Supplemental Information 6 Sequences of specific primers

10.7717/peerj.21299/supp-7 Supplemental Information 7 The protein sequence of 174 CpWRKY TFs

10.7717/peerj.21299/supp-8 Supplemental Information 8 Physicochemical properties of 174 CpWRKY TFs

10.7717/peerj.21299/supp-9 Supplemental Information 9 The structural features of motif 1–10

10.7717/peerj.21299/supp-10 Supplemental Information 10 The positions of motifs 1–10 in the 174 CpWRKYs

10.7717/peerj.21299/supp-11 Supplemental Information 11 The sequence positions of the main domains of the 174 CpWRKY members

10.7717/peerj.21299/supp-12 Supplemental Information 12 The secondary structure of 21 CpWRKY proteins

10.7717/peerj.21299/supp-13 Supplemental Information 13 List of the Ka, Ks, and Ka/Ks in *CpWRKY* segmental duplication gene pairs

10.7717/peerj.21299/supp-14 Supplemental Information 14 List of the *CpWRKY* tandem duplication gene pairs

10.7717/peerj.21299/supp-15 Supplemental Information 15 Tissue-specifically highly expressed *CpWRKY* genes

10.7717/peerj.21299/supp-16 Supplemental Information 16 Functional annotation of the target genes regulated by seven CpWRKY proteins

10.7717/peerj.21299/supp-17 Supplemental Information 17 GO enrichment of downstream genes regulated by Seven CpWRKY Proteins

10.7717/peerj.21299/supp-18 Supplemental Information 18 KEGG enrichment of downstream genes regulated by Seven CpWRKY Proteins

10.7717/peerj.21299/supp-19 Supplemental Information 19 Functional annotation of proteins interacted by fourteen CpWRKY proteins

10.7717/peerj.21299/supp-20 Supplemental Information 20 RNA/cDNA concentration

10.7717/peerj.21299/supp-21 Supplemental Information 21 Original transcriptome data of 174 *CpWRKY* genes

10.7717/peerj.21299/supp-22 Supplemental Information 22 MIQE checklist

## Additional Information and Declarations

### Data Availability

The following information was supplied regarding data availability: The raw data is available in the Supplemental Files.
