# Supplementary figures and images for: Genome-wide identification of WRKY members in elephant grass (Cenchrus purpureus) and its expression profiling under aluminum stress
Source: PeerJ. 2026 Jun 4;14:e21299. doi: 10.7717/peerj.21299 (PMC13242747; doi:10.7717/peerj.21299)

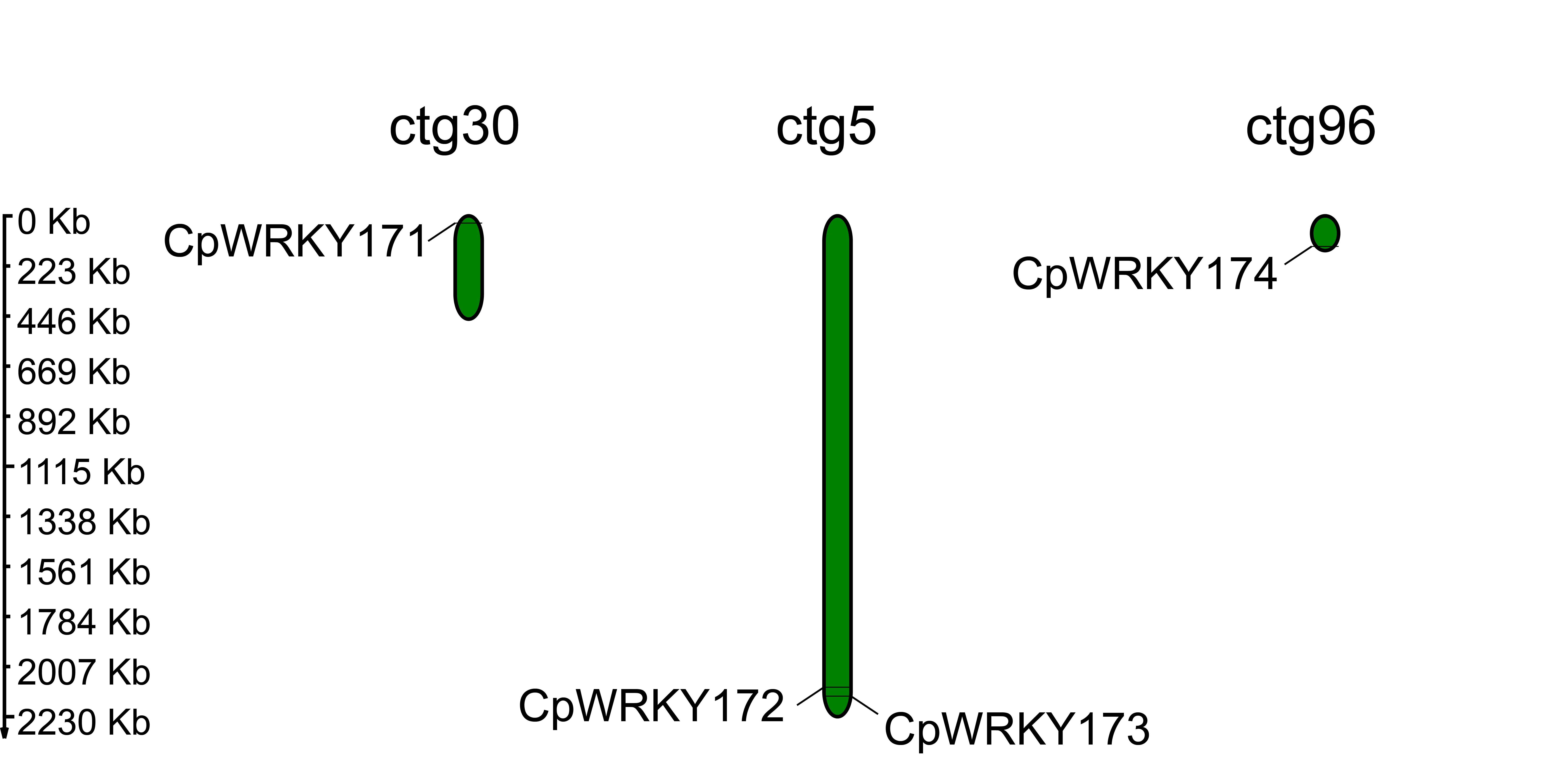

Supplement: Supplemental Information 1 [file peerj-14-21299-s001.png]

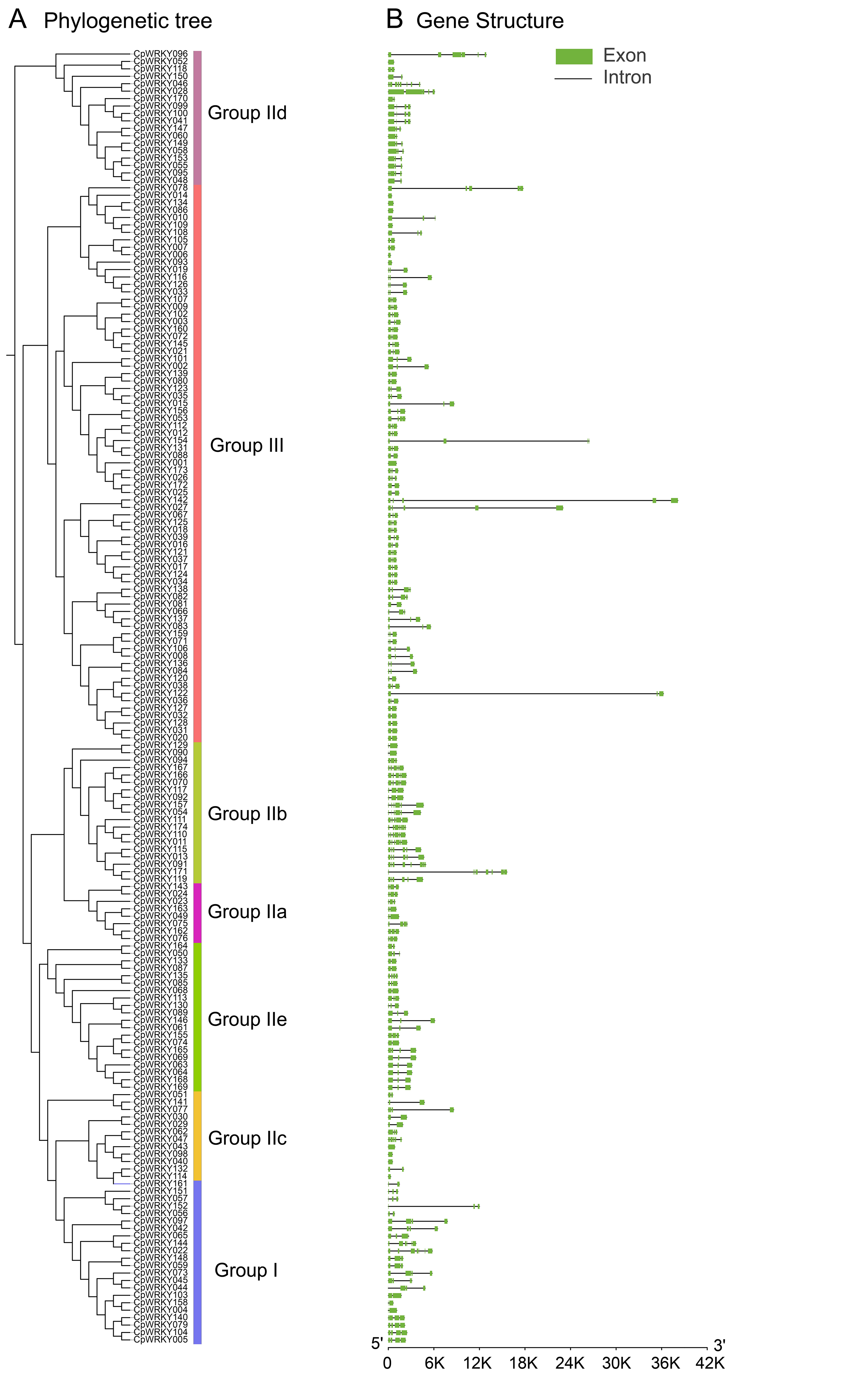

Supplement: Supplemental Information 2 [file peerj-14-21299-s002.png]

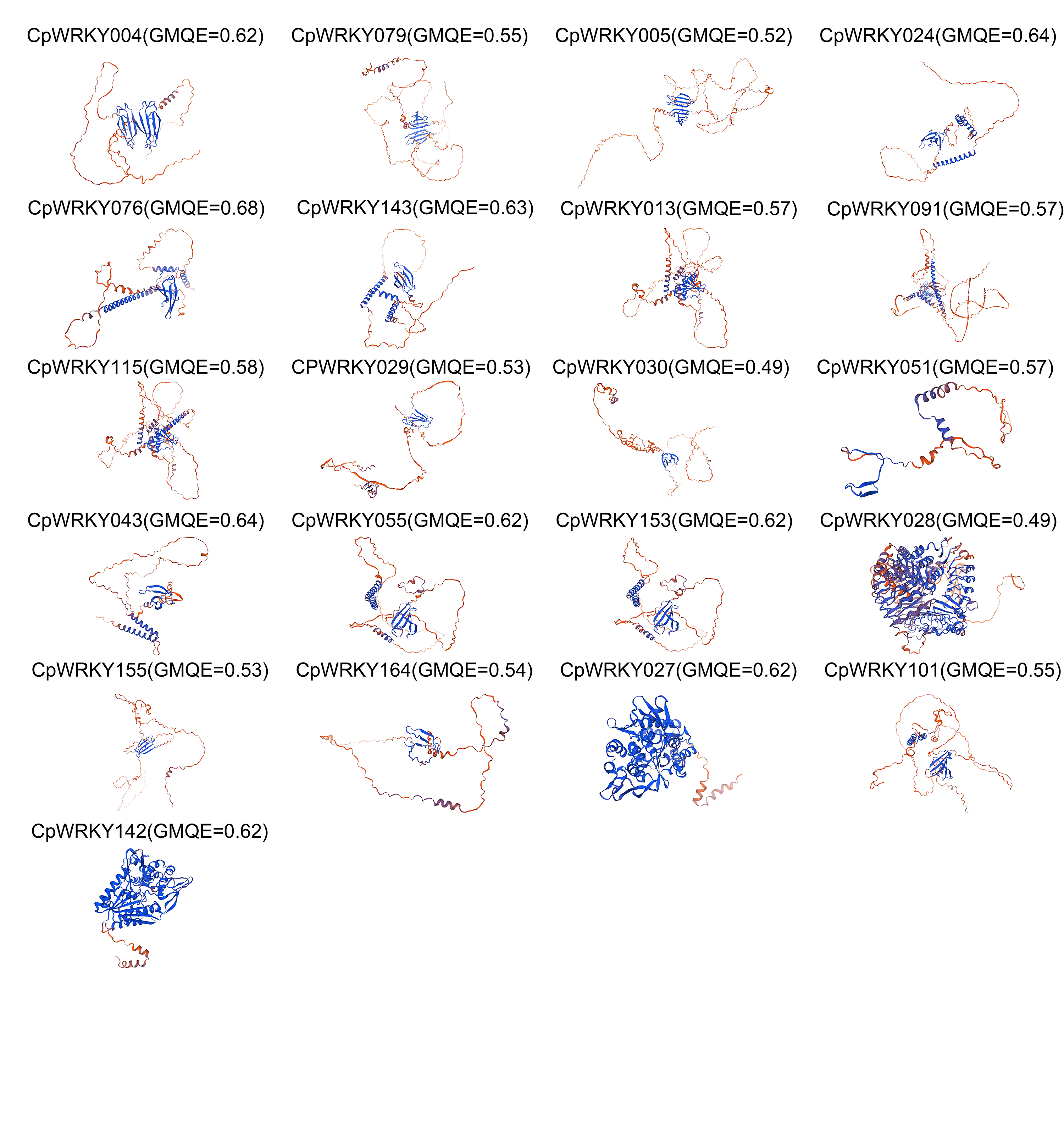

Supplement: Supplemental Information 3 — The model quality was evaluated using GMQE. [file peerj-14-21299-s003.png]

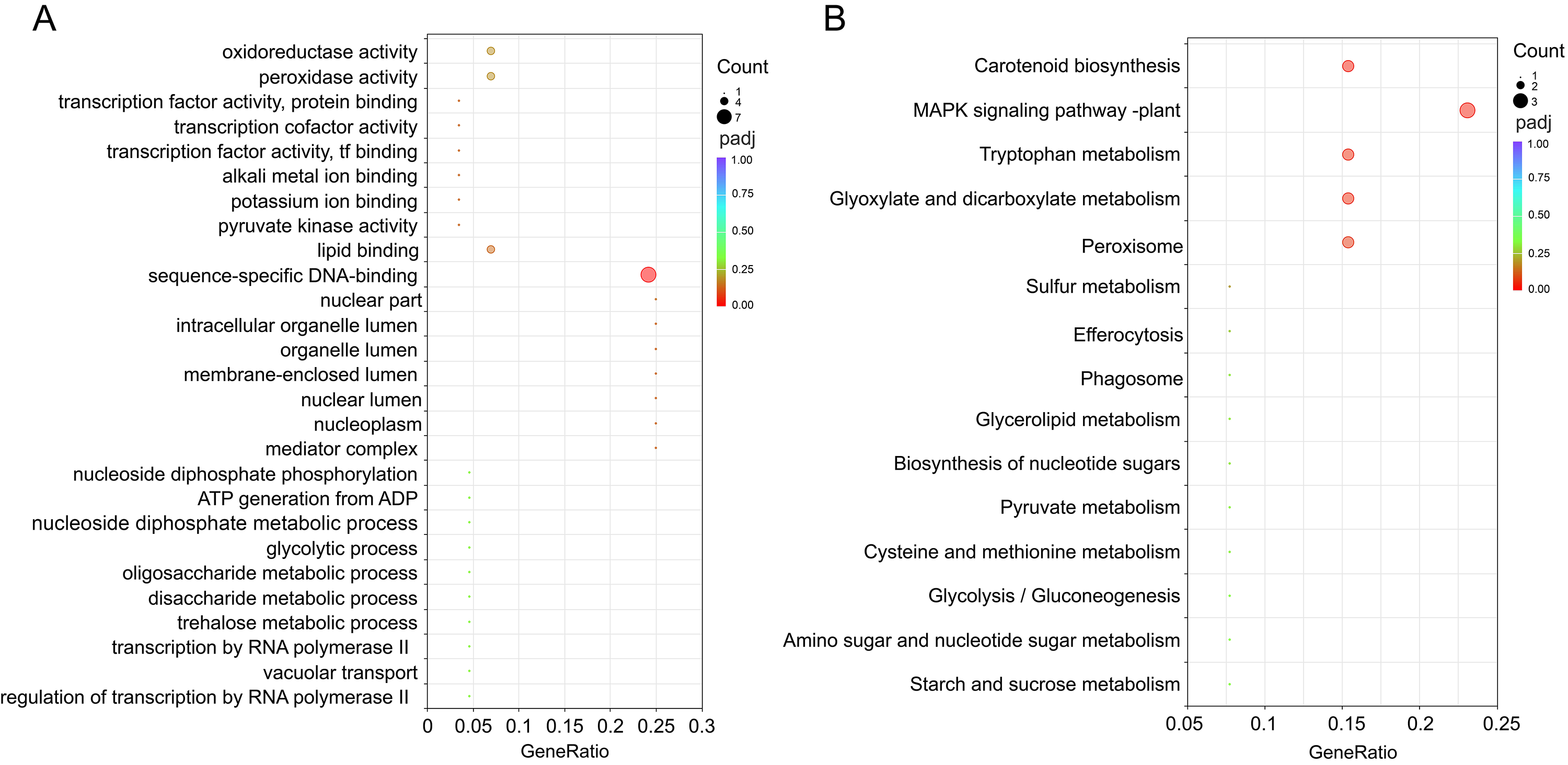

Supplement: Supplemental Information 4 — (A) GO enrichment analyses of downstream elephant grass genes potentially regulated by CpWRKY TFs. (B) KEGG enrichment analyses of downstream elephant grass genes potentially regulated by CpWRKY TFs. [file peerj-14-21299-s004.png]
